# The Abundant and Unique Transcripts and Alternative Splicing of the Artificially Autododecaploid London Plane (*Platanus* × *acerifolia*)

**DOI:** 10.3390/ijms241914486

**Published:** 2023-09-23

**Authors:** Xu Yan, Xiyan Chen, Yangyang Li, Yuhan Li, Fei Wang, Jiaqi Zhang, Guogui Ning, Manzhu Bao

**Affiliations:** National Key Laboratory for Germplasm Innovation and Utilization of Horticultural Crops, College of Horticulture and Forestry Sciences, Huazhong Agricultural University, Wuhan 430070, China; ianmooneyx@webmail.hzau.edu.cn (X.Y.); jiaqizhang@mail.hzau.edu.cn (J.Z.);

**Keywords:** alternative splicing (AS), artificially autododecaploid, few-flower, Oxford Nanopore Technologies (ONTs), *Platanus*, transcription

## Abstract

Transcription and alternative splicing (AS) are now appreciated in plants, but few studies have examined the effects of changing ploidy on transcription and AS. In this study, we showed that artificially autododecaploid plants of London plane (*Platanus* × *acerifolia* (Aiton) Willd) had few flowers relative to their hexaploid progenitors. Transcriptome analysis based on full-length Oxford Nanopore Technologies (ONTs) and next-generation sequencing (NGS) revealed that the increased ploidy level in *P.* × *acerifolia* led to more transcribed isoforms, accompanied by an increase in the number of isoforms per gene. The functional enrichment of genes indicated that novel genes transcribed specifically in the dodecaploids may have been highly correlated with the ability to maintain genome stability. The dodecaploids showed a higher number of genes with upregulated differentially expressed genes (DEGs) compared with the hexaploid counterpart. The genome duplication of *P.* × *acerifolia* resulted mainly in the DEGs involved in basic biological pathways. It was noted that there was a greater abundance of alternative splicing (AS) events and AS genes in the dodecaploids compared with the hexaploids in *P.* × *acerifolia*. In addition, a significant difference between the structure and expression of AS events between the hexaploids and dodecaploids of *Platanus* was found. Of note, some DEGs and differentially spliced genes (DSGs) related to floral transition and flower development were consistent with the few flower traits in the dodecaploids of *P.* × *acerifolia*. Collectively, our findings explored the difference in transcription and AS regulation between the hexaploids and dodecaploids of *P.* × *acerifolia* and gained new insight into the molecular mechanisms underlying the few-flower phenotype of *P.* × *acerifolia*. These results contribute to uncovering the regulatory role of transcription and AS in polyploids and breeding few-flower germplasms.

## 1. Introduction

*Platanus*, commonly known as the Plane tree, is one of the most important urban tree species worldwide, earning the title of the “king of street trees” [1]. Among the *Platanus* species, London plane (*Platanus* × *acerifolia*, with the synonym name of *Platanus* × *hispanica* Münchh.) is recorded as a hybrid of *Platanus occidentalis*, which is native to the eastern United States, and *Platanus orientalis*, which is native to Southwest Asia and Southeast Europe [1,2,3]. All *Platanus* species, including *P*. × *acerifolia*, are considered to be hexaploid species [4,5]. *P.* × *acerifolia* is the most widely used *Platanus* for urban forestry due to its fast growth, wide crown, adaptability to a range of environmental conditions, and tolerance to pollution [6,7]. However, *P.* × *acerifolia* does have several negative features, such as the release of large amounts of pollen in spring, the shedding of fruit hairs, and the dropping of fruit from late autumn to early summer, which not only affects human health but also pollutes the environment [8,9]. Breeding strategies to produce flowerless cultivars of *P.* × *acerifolia* offer potential solutions to these issues.

Artificial induction of polyploidy is a commonly used method for achieving plant genetic sterility and has been successfully applied to the production of seedless fruits or sterile phenotypes in various horticultural plants [10,11,12]. Previous research showed that polyploids commonly undergo genetic and epigenetic changes, and to maintain new gene dosage balance, this leads to differences in expression patterns between different ploidies [13,14,15,16,17]. Despite great interest, transcriptional and post-transcriptional differences between different ploidies are still in the preliminary exploration stage [18]. Previously, several polyploids were obtained based on the wild-type *P.* × *acerifolia* (2*n* = 42) through a colchicine treatment [19]. Among these, an autopolyploid “Hua Nong Qing Long” exhibits a phenotype that is similar to hexaploid plants in terms of nutritional growth but has far fewer flowers for its reproductive growth. Differences in flower transition between different ploidies reveal differential expression patterns, but the molecular mechanisms underlying the unique few-flower phenotype in polyploid *Platanus* remain an outstanding question.

Polyploidization leads to the disruption of meiotic and mitotic processes, as well as perturbations among cellular components, resulting in the transcriptional activation of new genes [20,21,22,23]. Additionally, AS is a widespread phenomenon in eukaryotes that generates different mature mRNAs from a single mRNA precursor and is involved in encoding different proteins from a single gene [24,25,26]. During polyploidization, there are changes in the composition, abundance, and activity of splicing factors that affect alternative splicing (AS) in polyploid wheat and cotton [27,28,29]. Studies showed that AS is more complex in polyploid *Brassica napus* due to an increase in the number of introns that provide more exon junctions [30]. However, the impact of differential new transcriptional and AS events on *Platanus* polyploid remains unclear.

Nanopore long-read transcriptome sequencing allows for the sequencing of full-length RNA molecules, avoiding the read length limitations of short-read sequencing technologies [31,32]. The obtained full-length transcripts include complete exons and splicing sites, providing comprehensive and accurate information on alternative splicing [33,34,35]. Nanopore long-read transcriptome sequencing provides the ability to accurately analyze transcriptional and alternative splicing differences between polyploids and diploids of *Nicotiana tabacum*, *Brassica napus*, and *Helianthus annuus* [35,36,37].

In this study, we developed an effective workflow for deciphering gene splicing and expression using Oxford Nanopore Technologies (ONTs) and NGS sequencing in organisms without reference genome analysis. Through transcriptome profiling from different tissues and developmental stages of subpetiolar buds of *P.* × *acerifolia* hexaploid and artificial *P.* × *acerifolia* autododecaploid, we revealed the polyploid-specific transcripts and identified AS differences between different ploidies. We characterized the impact of polyploidy on transcriptional and post-transcriptional regulation and elucidated the molecular mechanism underlying the unique floral transition phenotype in *P.* × *acerifolia* polyploids. Overall, this study provides novel insights into transcriptional and post-transcriptional regulation in *P.* × *acerifolia* and plant polyploids and holds great significance for future molecular breeding of *P.* × *acerifolia*.

## 2. Results

### 2.1. Dodecaploid Plants Exhibited a Distinct Morphology

**Figure 1:**
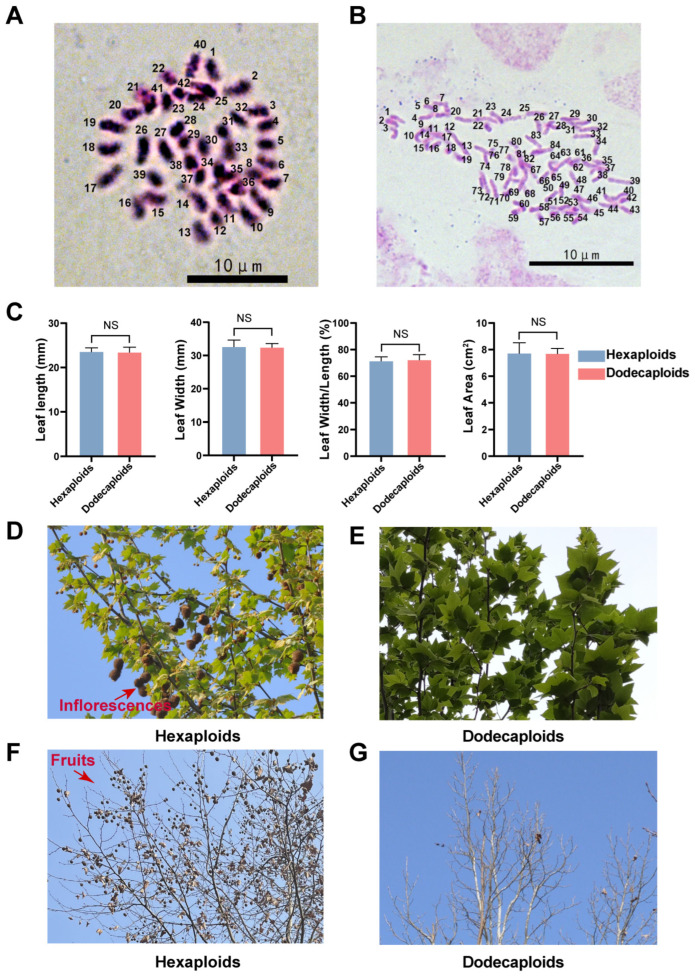
Comparison of ploidy and morphological characteristics between hexaploid and dodecaploid *P.* × *acerifolia*. (**A**) The chromosome numbers of the wild-type hexaploid *P*. × *acerifolia*; (**B**) the chromosome numbers of the autododecaploids *Platanus* “Hua Nong Qing Long”; (**C**) a comparison of leaf traits (leaf length, leaf width, leaf length-to-width ratio, and leaf area) between hexaploids and dodecaploids, “NS” indicates no significant difference; (**D**) an image of flowering stage in hexaploid *P.* × *acerifolia*; (**E**) an image of flowering stage in dodecaploid *P.* × *acerifolia*; (**F**) an image of fruiting stage in hexaploid *P.* × *acerifolia*; (**G**) an image of fruiting stage in dodecaploid *P.* × *acerifolia*.

The ploidy level of *P.* × *acerifolia* “Hua Nong Qing Long” was verified via chromosome counting (Figure 1A,B). Under normal growth conditions, dodecaploid plants exhibited a similar plant morphology to hexaploid counterparts, except for the flower number. There was no significant difference in the leaf morphology between hexaploids and dodecaploids of *P.* × *acerifolia*, including leaf length, width, width-to-length ratio, and leaf area (Figure 1C). However, the dodecaploids had significantly fewer flowers and fruits than the hexaploids, which had a very high application value (Figure 1D–G).

### 2.2. Dodecaploids Produce More Genes and Isoforms in P. × acerifolia

To maximize the transcriptome coverage, we combined a total of 1604.93 million clean NGS reads from the hexaploid and autododecaploid samples and performed de novo transcriptome assembly (Supplementary Table S1). To obtain a maximal number of contigs, we employed a low threshold during the NGS short-read assembly and generated a total of 239,977 transcripts with an average contig length of 636.3 bp and an N50 of 836 bp after removing any redundancy (Supplementary Table S2). 

**Figure 2:**
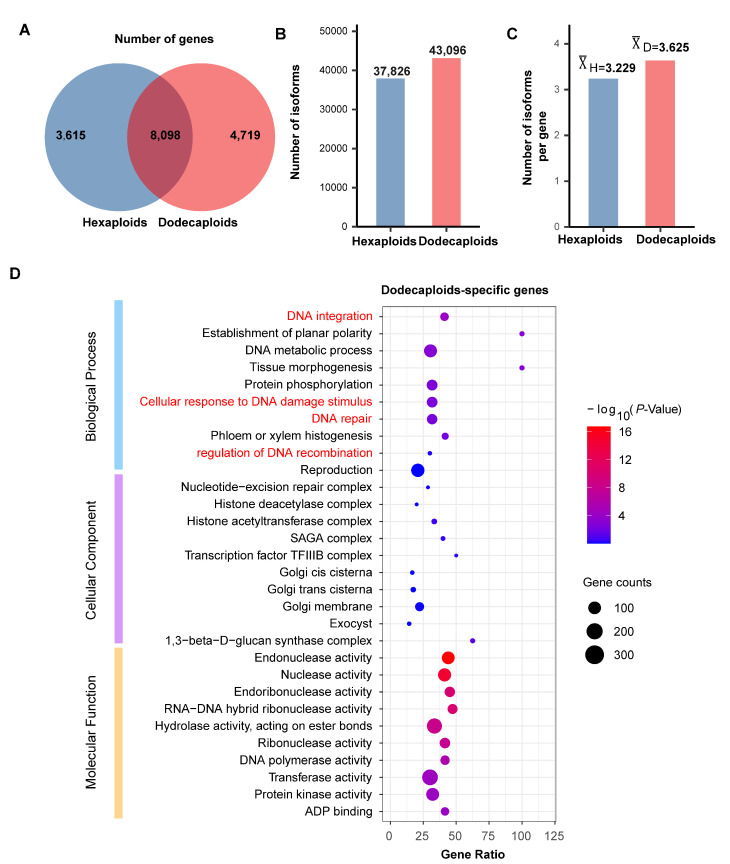
The genes and isoforms in hexaploids and dodecaploids of *P*. *× acerifolia*. (**A**) The number of genes in hexaploids and dodecaploids, respectively; (**B**) the number of isoforms in hexaploids and dodecaploids, respectively; (**C**) the number of isoforms per gene in hexaploids and dodecaploids; (**D**) GO term enrichment analysis of genes specifically transcribed in dodecaploids.

In the hexaploids and dodecaploids, 28,047,798 and 29,944,161 reads, respectively, were generated using ONT sequencing (Supplementary Table S3). After clustering the NGS-corrected ONT reads, we obtained 9,024,323 and 8,436,331 non-redundant, error-corrected full-length reads from the hexaploids and dodecaploids, respectively (Supplementary Table S4). Subsequently, the mapped longest NGS contigs were used as representative of genes (Figure S1) [38]. A total of 16,432 genes were mapped using unique ONT reads, of which, 3615 were specifically transcribed in hexaploids, 4719 were specifically transcribed in dodecaploids, and 8098 genes were transcribed in both the dodecaploids and hexaploids (Figure 2A and Supplementary Tables S5 and S6). The mapping result at the 99% identity level shows that 37,826 ONT reads from the hexaploids and 43,096 ONT reads from the dodecaploids were aligned with these genes (Figure 2B). Furthermore, an average of 3.229 isoforms per gene were observed in the hexaploids, while the dodecaploids exhibited an average of 3.625 isoforms per contig (Figure 2C). It is worth noting that the number of isoforms produced by ploidy-specific genes (1.430 in the hexaploids and 1.875 in the dodecaploids) was significantly lower than that produced by genes shared between the hexaploids and dodecaploids (4.033 in the hexaploids and 4.387 in the dodecaploids) in *Platanus* (Figure S2). Taken together, these results suggest that increased ploidy in *Platanus* resulted in the transcription of more genes and isoforms, as well as an increase in the number of isoforms per gene.

### 2.3. Dodecaploid-Specifically Transcribed Genes Associated with Chromosome Repair in P. × acerifolia

To investigate the differences in biological processes between the hexaploids and dodecaploids, we performed function annotation (Supplementary Table S7) and Gene Ontology (GO) term enrichment analysis using contigs transcribed specifically in the hexaploids, specifically in the dodecaploids, and contigs transcribed in both the dodecaploids and hexaploids. Interestingly, we found that genes transcribed specifically in the dodecaploids were functionally enriched in the GO terms “cellular response to DNA damage stimulus”, “DNA repair”, and “regulation of DNA recombination” (Figure 2D). These enriched genes were thought to play an important role in maintaining the stability of chromosomes.

We found that the enrichment results of genes transcribed in both the hexaploids and dodecaploids and genes transcribed specifically in the hexaploids were significantly enriched in essential plant life processes, such as “defense response”, “DNA integration”, “RNA processing”, “cellular component organization or biogenesis”, “photosynthesis”, and several metabolism processes, but there was no significant enrichment in the GO terms related to DNA repair (Figure 2D and Figure S3). These results indicated that the differences in transcription between the hexaploids and dodecaploids, especially the genes transcribed specifically in the dodecaploids, might be highly correlated with the ability to maintain genome stability.

### 2.4. Differentially Expressed Genes between Hexaploids and Dodecaploids

**Figure 3:**
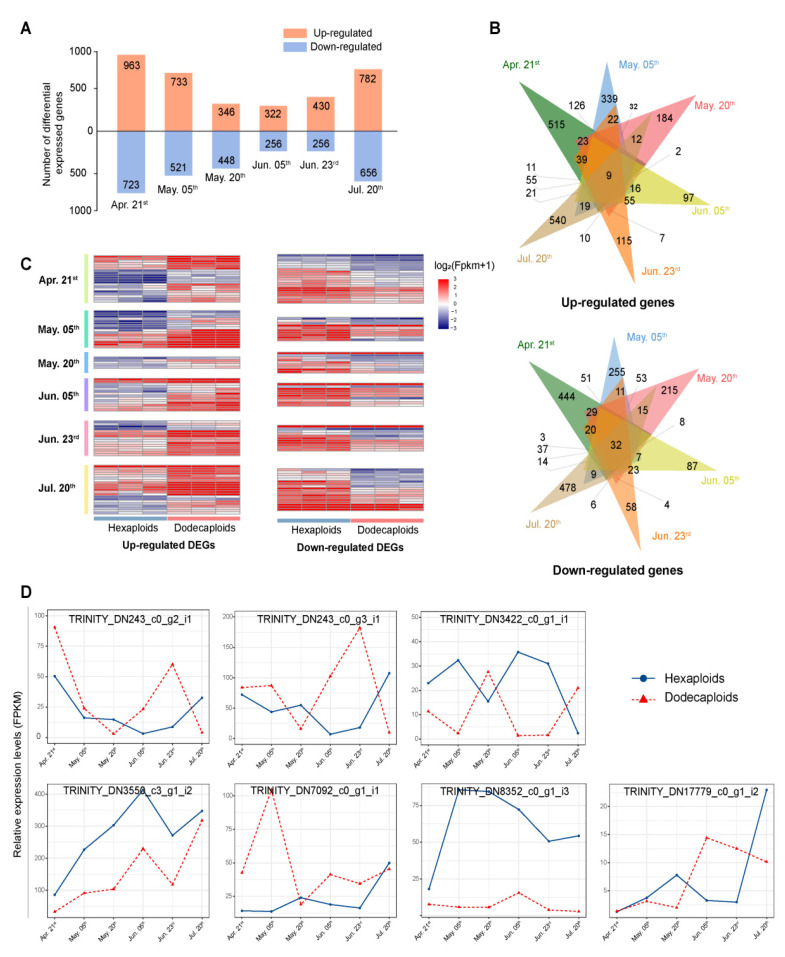
DEGs between hexaploids and dodecaploids in *P*. *× acerifolia*. (**A**) The number of DEGs between hexaploids and dodecaploids in stages 1–6 of floral transition, respectively; (**B**) the Venn diagrams of the number of upregulated and downregulated DEGs across the six stages of floral transition between hexaploids and dodecaploids; (**C**) the heatmap of DEGs related to flower development and floral transition; (**D**) the expression patterns of flowering-related significant DEGs between dodecaploids and hexaploids.

To investigate the difference in floral transition between the hexaploids and dodecaploids, we analyzed the differentially expressed genes (DEGs) of the floral transition with the six subpetiolar bud development stages using the longest ONT-mapped contigs of NGS as the reference (Supplementary Tables S8–S13). A total of 1686 (963 upregulated and 723 downregulated), 1254 (733 upregulated and 521 downregulated), 794 (346 upregulated and 448 downregulated), 578 (322 upregulated and 256 downregulated), and 1438 (782 upregulated and 656 downregulated) DEGs were identified in the stages 1–6 of floral transition in the dodecaploids, respectively, in comparison with the hexaploids (Figure 3A). Among the DEGs, 9 were upregulated, while 32 were downregulated in all six floral transition stages of the dodecaploids (Figure 3B). It was also found that the total amount of upregulated DEGs was greater than that of downregulated DEGs in the dodecaploids in five out of six comparisons of floral transition stages between the dodecaploids and hexaploids (Figure 3B). The dodecaploids exhibited a higher number of genes with upregulated DEGs compared with the hexaploid counterpart.

To investigate the role of genome doubling in the dodecaploids’ biological processes, we conducted a comprehensive analysis of DEGs that were differentially expressed in the six stages of subpetiolar buds during the floral transition between dodecaploids and hexaploids. The six stages of upregulated DEGs between the hexaploid and dodecaploid are enriched in different terms, and the most highly enriched terms of each of the six stages were “cell wall biogenesis”, “defense response”, “response to biotic stimulus”, “circadian regulation of gene expression”, “response to stimulus”, and “response to abiotic stimulus” (Figures S4–S10). The most highly enriched terms of the downregulated DEGs of each of the six stages were “cell wall organization or biogenesis”, “regulation of hormone”, “cutin biosynthetic process”, “RNA modification”, “mitochondrial RNA metabolic process”, and “phenylpropanoid biosynthetic process” (Figures S11–S16). These results suggest that genome duplication in *P.* × *acerifolia* mainly leads to the differential expression of genes involved in basic biological pathways, such as cell wall organization or biogenesis, stress response, hormone signaling, and circadian rhythms. 

Of note, although there was no significant enrichment of differentially expressed genes in flower development and flowering-related pathways at all six stages of subpetiolar bud development, a large number of flower-development- and flowering-related genes were still found to be differentially expressed between hexaploids and dodecaploids. Cumulatively, up- and downregulated DEGs in dodecaploids reached 60 and 49 flower-development- and flowering-related genes, respectively (Figure 3C). Among these flower-related DEGs, seven genes were significantly differentially expressed in at least four floral transition stages between the dodecaploids and hexaploids, and all seven genes showed different expression trends or consistently extreme differences in the dodecaploids compared with the hexaploids (Figure 3D). Both TRINITY_DN243_c0_g2_i1 and TRINITY_DN243_c0_g3_i1 were annotated as *LHY-like* (*LATE ELONGATED HYPOCOTYL*) genes and showed similar expression trends: the dodecaploid significantly increased transcription from 20 May to 23 June and decreased in July, whereas the hexaploid showed the opposite trend and much less expression than the dodecaploid. TRINITY_DN3422_c0_g1_i1 (annotated to *COR27*; *Cold-regulated protein 27*), TRINITY_DN7092_c0_g1_i1 (annotated to *BBX32*; *B-box zinc finger protein 32-like*), and TRINITY_DN17779_c0_g1_i2 (annotated to *NAC029*; *NAC transcription factor 29-like*) showed different expression trends between the dodecaploids and hexaploids, and there were significant differences at several stages. In particular, *COR27* showed the opposite expression trend in the dodecaploids compared with the hexaploids, which is an important transcription factor involved in the transcription of *TOC1* and *PRR5* that represses their transcription and regulates flowering time [39,40]. In addition, TRINITY_DN3550_c3_g1_i2, which corresponds to the zinc finger protein *COL5* (*CONSTANS-LIKE 5*), and TRINITY_DN8352_c0_g1_i3, which was identified as the *AP1* (*APETALA 1*), exhibited a markedly lower level of expression in the dodecaploids compared with the hexaploids throughout all stages of subpetiolar bud development. Previous studies regarded their homologs as pivotal genes involved in flower development and the process of flowering [39,40,41,42,43,44,45,46,47,48,49]. These results imply that the flowerless phenotype of the dodecaploid may be associated with the differential expression of genes related to flower development and flowering.

### 2.5. Differentially Alternative Splicing Events between Hexaploids and Dodecaploids in P. × acerifolia

**Figure 4:**
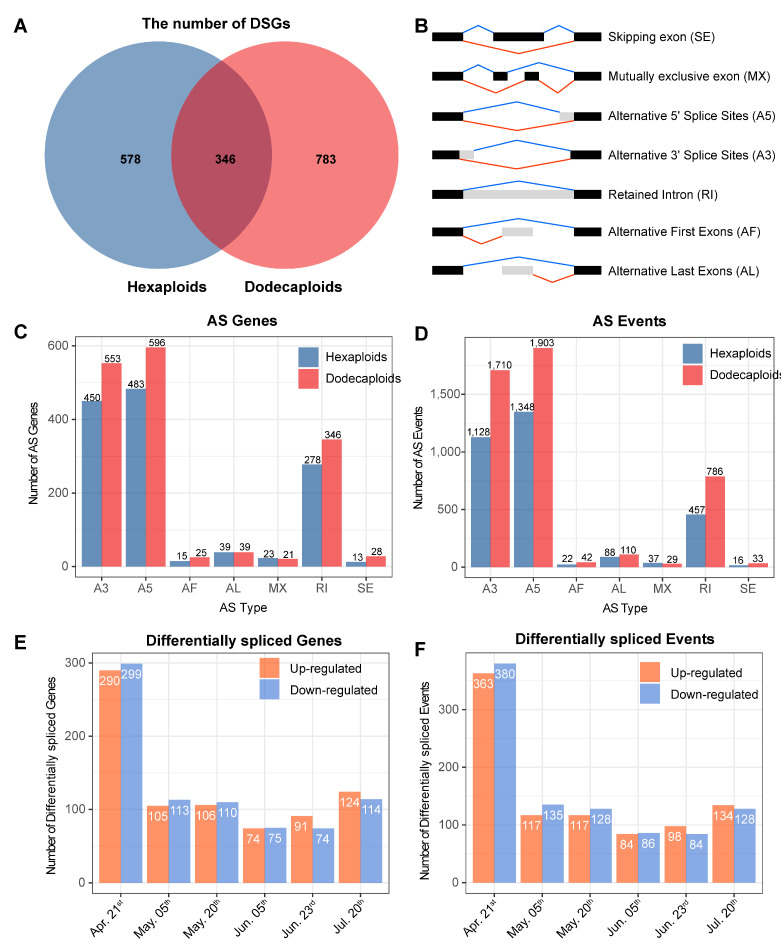
The differences of AS genes and AS events between hexaploids and dodecaploids in *P*. *× acerifolia*. (**A**) The Venn diagrams of number of AS genes; (**B**) the schematic structure of the AS types analyzed in this study; (**C**) number of AS genes across different AS types in hexaploids and dodecaploids, respectively; (**D**) number of AS events across different AS types in hexaploids and dodecaploids, respectively; (**E**) the number of upregulated and downregulated differentially spliced genes between dodecaploids and hexaploids in 6 floral transition stages; (**F**) the number of upregulated and downregulated differentially spliced events between dodecaploids and hexaploids in 6 floral transition stages.

Our previous study showed that total AS genes accounted for approximately 10.39% (1707) of all genes, with 783 AS genes present only in the dodecaploids, 578 AS genes present only in the hexaploids, and only 346 AS genes present both before and after the genome doubling (Figure 4A). Specifically, 3096 AS events corresponded to 925 AS genes in the hexaploids, and 4613 AS events corresponded to 1130 AS genes in the dodecaploids. The distributions of AS types in the hexaploids and dodecaploids were comparable; that is, A5 (alternative 5’ splice sites) was the most abundant (41–43%) AS event, followed by A3 (alternative 3’ splice sites) (36–37%), and RI (retained intron) (15–17%), while the remaining four alternative splicing events—alternative first exons (AFs), alternative last exons (ALs), skipping exon (SE), and mutually exclusive exons (MXs)—accounted for only 5–6% of the AS events (Figure 4B–D). Collectively, these results showed that there were more AS events and AS genes in the dodecaploids than in the hexaploids, and that a large number of AS genes were different from hexaploids, suggesting that AS in *P.* × *acerifolia* was altered by increased ploidy.

To explore the AS changes in response to ploidy change, we identified ploidy-responsive AS events, which refer to the splicing isoforms of a gene that showed differential expression changes between the hexaploids and dodecaploids (Supplementary Tables S14–S19). In total, 1854 (913 upregulated and 941 downregulated) AS events from 677 genes responded to the ploidy changes in the dodecaploid *Platanus* (Figure 4E). Specifically, 363, 117, 117, 84, 98, and 134 AS events were characterized as upregulated in stages 1–6 of the dodecaploid bud development, respectively, whereas the corresponding number for downregulated was 380, 135, 128, 86, 84, and 128, respectively (Figure 4F). Notably, these AS events and genes exhibited significantly higher number levels in April than in the other stages of floral transition, suggesting that the AS events of the early stage of floral transition (April) are likely to play a critical role in the transcriptional regulation difference between the hexaploids and dodecaploids. 

To investigate the role played by differential splicing between the hexaploids and dodecaploids in the floral transition of *P.* × *acerifolia*, we analyzed the enrichment of terms in the biological process of differential spliced genes (DSGs) between ploidies during the six stages of subpetiolar bud development. The results revealed that April was the period with the highest number of AS events during bud development, and the enrichment of DSGs in April showed that “cellular modified amino acid metabolic process” was the most highly enriched term (e.g., “peptidyl-proline hydroxylation” and “hydroxyproline metabolic process”), followed by “endomembrane system organization” and “protein hydroxylation” (Figure S17). The subsequent five periods were mostly enriched in similar terms, mainly including “regulation of stem cell population maintenance”, “regulation of immune system process”, “mucilage metabolic process”, “seed coat development”, “shoot apical meristem development”, “RNA splicing”, “removal of nonhomologous ends”, “pyrimidine dimer repair by nucleotide-excision repair”, and “vegetative phase change” (Figures S18–S22). 

**Figure 5:**
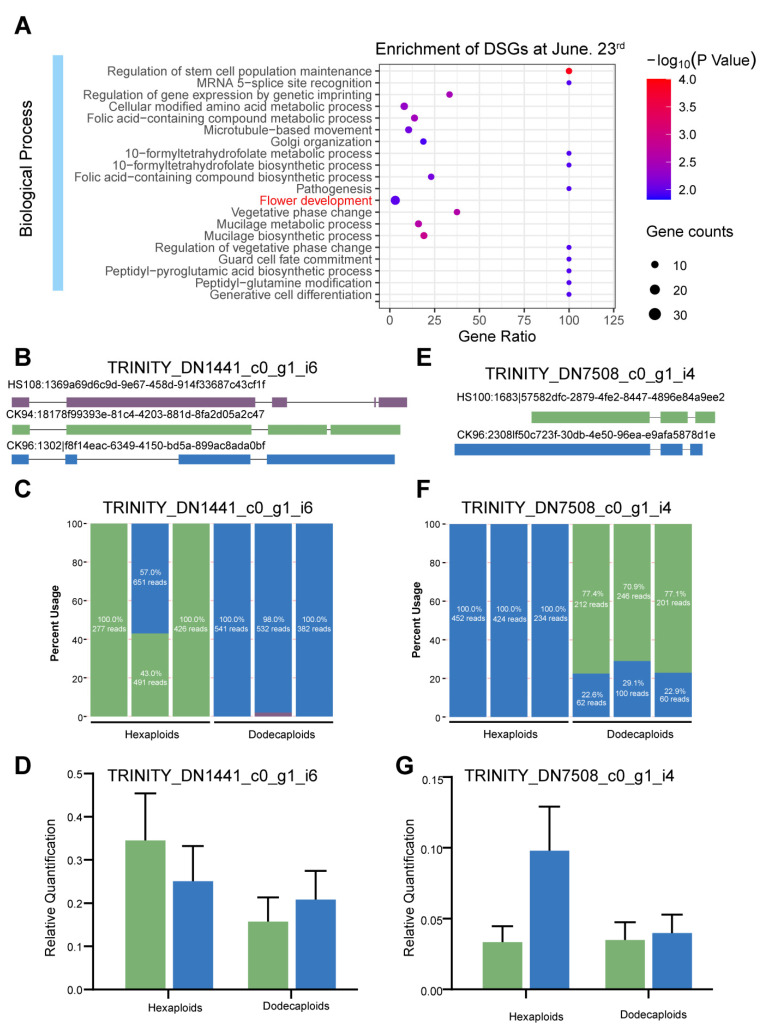
The DSGs related to flower development between hexaploids and dodecaploids. (**A**) The DSGs between hexaploids and dodecaploids were enriched in “flower development” on 23 June; the schematic (**B**), RNA-seq expression profile (**C**), and qRT-PCR (**D**) of AS events in TRINITY_DN1441_c0_g1_i6; the schematic (**E**), RNA-seq expression profile (**F**), and qRT-PCR (**G**) of AS events in TRINITY_DN7508_c0_g1_i4.

Of note, the DSGs on 23 June were significantly enriched in flower development, including nine genes identified as flower-development-related genes (Figure 5A and Figure S23). These genes revealed significant differences in expression and alternative splicing between the hexaploids and dodecaploids, including *LUH* (*LEUNIG_HOMOLOG*; TRINITY_DN978_c0_g1_i8), which was reported to repress the transcription of *AGAMOUS* and abort the flower [50,51]; *GCN5* (TRINITY_DN7508_c0_g1_i4), which was reported to affect inflorescence meristem and stamen development [52,53]; *DCAF1* (TRINITY_DN7245_c0_g1_i2), which was reported to affect flower development and embryogenesis [54]; *CYP78A5* (TRINITY_DN6315_c0_g1_i6), which was associated with floral organogenesis and reduced fertility [55]; *SAP130A* (TRINITY_DN6048_c0_g1_i1), which was required for pollen and ovule development [56]; *FLXL4* (TRINITY_DN37958_c0_g1_i3), which was involved in *FLC* activation and early flowering [57,58]; *TFIIS* (TRINITY_DN32671_c0_g1_i19), which was related to early flowering and seed dormancy [59]; *PGI1* (TRINITY_DN10518_c0_g1_i16), whose mutant lead to late flowering [60]; and *FRL1* (TRINITY_DN1441_c0_g1_i6), whose functional allele was required for the late-flowering-time phenotype caused by the *FRIGIDA* functional allele [61].

To confirm the flower-related AS events between the hexaploids and dodecaploids identified using RNA sequencing, we further validated the predicted AS patterns using the reverse transcription (RT)-PCR of isoforms. As expected, two investigated DSGs, including *FRL1* and *GCN5*, showed consistent AS patterns with their profiles revealed using RNA-seq data (Figure 5B,E). This further confirmed the accuracy of our bioinformatic analysis. In the dodecaploids, the isoform CK96:1302lf8f14eac-6349-4150-bd5a-899ac8ada0bf of FRL1 (TRINITY_DN1441_c0_g1_i6) showed a higher expression ratio compared with the CK94:18178f99393e-81c4-4203-881d-8fa2d05a2c47 isoform, while an opposite trend was observed in hexaploids (Figure 5C,D). Likewise, the isoform CK96:23081f50c723f-30db-4e50-96ea-e9afa5878d1e of GCN5 (TRINITY_DN7508_c0_g1_i4) was expressed in a similar proportion to that of HS100:1683157582dfc-2879-4fe2-8447-4896e84a9ee2 expressed in the dodecaploids, while the former isoform was expressed more than twice as much as the latter isoform in hexaploids (Figure 5F,G). These results indicate that the increase in ploidy led to substantial changes in differential splicing and flower morphology.

## 3. Discussion

Polyploidization commonly occurs in plants and is considered a major driving force in plant evolution [12,62,63]. Many studies have demonstrated that polyploidy can lead to trait alterations such as organ size, and environmental adaptability, offering potential for plant breeding [64,65]. In this study, we observed no significant differences in leaf traits (leaf length, leaf width, leaf length-to-width ratio, leaf area) between the dodecaploid and hexaploid plants. While many polyploid plants exhibit distinct leaf morphologies [66,67], the dodecaploids showed fewer flowers than hexaploids in *Platanus*. As one of the most widely used urban trees worldwide, dodecaploid *P.* × *acerifolia* holds immense potential in the landscape industry. Future extensive work should be conducted to assess the potential of dodecaploid plants as excellent urban trees for trait improvement.

The cultivation of polyploid plants with desirable traits has a history of over 100 years [68,69,70]. However, our understanding of the molecular mechanisms underlying transcriptional differences between polyploid and diploid plants remains highly limited [18]. Polyploidy plays a major role in driving species formation and facilitating the evolution of novel gene functions. In our study, we found that the autododecaploid *P.* × *acerifolia* exhibited increased transcription of genes and isoforms and there was a higher number of isoforms per gene. Similarly, previous studies showed a higher incidence of changes in AS patterns in the natural heteropolyploid *Brassica napus* compared with its diploid ancestor [37]. It is hypothesized that the combination of regulatory factors inherited from different parental sources, together with epigenetic modifications following genome duplication, contribute to the amplification of transcribed gene isoforms in allopolyploids [25,30]. However, the specific molecular mechanisms responsible for the generation of additional isoforms in autopolyploids remain elusive.

The presence of multiple chromosome copies following plant polyploidization disrupts the typical meiotic process observed in diploids [71,72,73]. In newly formed polyploids, the sudden appearance of multiple homologous chromosomes can lead to mixed interactions and erroneous chromosome segregation during meiosis [63]. However, the prevalence of stable and fertile polyploids in both cultivated and wild plants suggests that specialized meiotic programs may evolve following whole-genome hybridization to mitigate such interactions and subsequent meiotic irregularities [13]. In our current investigation, we found that genes specifically transcribed in the *P.* × *acerifolia* dodecaploids were functionally enriched in GO terms associated with the maintenance of chromosome stability, including “cellular response to DNA damage stimuli”, “DNA repair”, and “regulation of DNA recombination”. Interestingly, genes specifically expressed in *Platanus* hexaploids or selectively spliced in the dodecaploids were not found to be associated with DNA repair functions. Therefore, the preferential activation of genes newly transcribed in the dodecaploid *Platanus*, rather than selective splicing resulting from polyploidy, is proposed as a mechanism to prevent chromosome missegregation due to polyploidy. However, the underlying mechanisms by which these DNA repair genes exert their functions remain largely unknown and require further comparative proteomic and cytological analyses of polyploid and hexaploid progenitor cells.

Differences in gene expression between different ploidy plants can help us understand the effects of polyploidization on organisms and its underlying mechanisms. In this study, we found a large number of differentially expressed genes (DEGs) at each of the six stages of floral transition in *Platanus*. In contrast, for both of the six stages, only 9 genes were upregulated and 32 genes were downregulated in the dodecaploids. DEGs between ploidy levels were enriched in different functional terms during each stage of *P.* × *acerifolia* floral transition. These results indicate that the differential genes induced by a ploidy increase vary greatly at each stage of *P.* × *acerifolia* floral transition. Ploidy increase in *P.* × *acerifolia* primarily leads to changes in cell wall organization, stress response, hormone signaling, and circadian rhythm, which may contribute to the enhanced environmental adaptability of dodecaploid plants. Our findings are consistent with recent studies where gene expression differences between different ploidy plants were found to enhance stress tolerance, highlighting the importance of gene expression changes for the survival of newly formed polyploids in harsh environments [74,75,76].

Although the critical role of alternative splicing (AS) in eukaryotes, including plants, has been recognized [77,78,79,80], there is a paucity of studies that analyzed the effect of polyploidy on AS. Our study showed that the number of AS events that occur in the dodecaploids was 150% higher than in the hexaploids. Furthermore, we found that only a small number of alternatively spliced genes (346) were shared between the dodecaploids and hexaploids, suggesting that the depth of AS was involved in shaping the life processes and traits of the polyploids. Similar patterns of AS in polyploids were observed in many other plants, such as wheat, Brassica, and cotton [27,28,30]. This could be attributed to an increase in the number of introns in polyploid plants, providing more exon junctions and resulting in a greater potential for gene regulation and protein diversity in polyploids.

The floral transition, which is a pivotal event in the life cycle of angiosperms, is regulated by intricate molecular networks and environmental cues [81,82]. In our study, we observed differential upregulation and downregulation of 60 and 49 genes, respectively, which were potentially associated with flowering transition and flower development in dodecaploid *P.* × *acerifolia*. Among these genes, some are well-characterized and known to be involved in flowering time and flower development, including *LHY-like*, *COR27*, *BBX32*, *NAC029*, *COL-5*, and *AP1*. These flower-regulated genes in dodecaploids exhibit distinct expression patterns compared with hexaploids and show significant differences at multiple development stages. Notably, *AP1* is required for the subsequent transition from an inflorescence meristem to a floral meristem, as well as for the normal development of sepals and petals [43,48]. The overexpression of *AP1* leads to early flowering and the transformation of apical and lateral shoots into flowers [48]. Increased expression of *COL5* elevates *FT* expression levels, resulting in early flowering [44]. *LHY* inhibits the transcription of *APRR1*, *CCA1*, and *TOC1*, leading to late flowering [45,46]. *COR27* delays flowering by suppressing the transcription of *TOC1*, *PRR5*, *ELF4*, and cold-responsive genes [39,40]. *BBX32* causes late flowering in *Arabidopsis* [47]. *NAC029* affects floral organ development in *Arabidopsis* [49]. Hence, it can be reasonably inferred that the differential expression of these flowering-transition- and flower-development-related genes may contributed to the reduced-flowering phenotype observed in the dodecaploids of *P.* × *acerifolia*. In this study, we also discovered some atypical genes associated with flowering time and flower development that show differential alternative splicing in the dodecaploid *P.* × *acerifolia*. These DSGs have been less studied in the context of flowering time and flower development; however, their limited investigation does not diminish their potential importance in these processes. LUH, which interacts with LEUNIG (LUG) and SEUSS (SEU), plays a role in inhibiting *AGAMOUS* transcription and leads to the failure of floral organ development [50,51]. *GCN5* was reported to affect inflorescence meristem and stamen development [52,53]. *DCAF1* was implicated in flower development and embryogenesis [54]. *CYP78A5* is associated with floral organogenesis and reduced fertility [55]. *SAP130A* is essential for pollen and ovule development [56]. *FLXL4* is involved in *FLC* activation and flowering time control [57,58]. *TFIIS* is associated with flowering time and seed dormancy [59]. *PGI1* affects flower initiation [60], while *FRL1* is known to function in flowering time control [61,83,84]. These results suggest that alternative splicing may contribute to transcriptome reprogramming and may serve as an important molecular mechanism in the regulation of flowering and flower development in *P.* × *acerifolia*. However, further experimental investigations are required to draw explicit conclusions regarding its biological relevance in *P.* × *acerifolia*.

## 4. Materials and Methods

### 4.1. Plant Materials

The plant materials for RNA-seq were collected from the subpetiolar buds of hexaploid wild-type *P.* × *acerifolia* and the flowerless artificial polyploid *P.* × *acerifolia* “Hua Nong Qing Long”, respectively, in anexperimental nursery at Huazhong Agricultural University (HZAU, Wuhan, China; 114.35 E, 30.48 N). The subpetiolar buds for wild-type and “Hua Nong Qing Long” were collected from April to July (including 21 April, 5 May, 20 May, 5 June, 23 June, and 20 July) with three replicates. For the ONT full-length sequencing, the subpetiolar buds from six stages of floral transition were mixed from wild-type and “Hua Nong Qing Long”. And subpetiolar buds from each period of wild-type and “Hua Nong Qing Long” were individually used for NGS sequencing with three replicates.

### 4.2. Ploidy Identification and Leaf Morphology Statistics

Ploidy levels were determined using young leaves with a known hexaploid plant as a control. The young roots of wild-type and “Hua Nong Qing Long” *P.* × *acerifolia* were sampled for chromosome counting [19] and photographed under an upright Leica DM6-B microscope (Leica Microsyetems, Wetzlar, Germany) at Huazhong Agriculture University in China.

The leaf morphologies were measured with three biological replicates for each phenotype and 10 technical replicates were performed for each biological replicate using ImageJ [85,86]. The *t*-tests were conducted and visualized using GraphPad Prism 9 (graphpad.com, accessed on 23 July 2023).

### 4.3. Sequencing of Transcriptomes

The RNA extraction was performed using the EASYspin polysaccharide and polyphenol plant total RNA extraction kit (Aidlab^®^, Beijing, China). Transcriptome samples, which were previously subjected to ultra-low-temperature freezing, were rapidly ground in liquid nitrogen, and RNA was extracted following the instructions provided with the corresponding kit. The extracted RNA was then subjected to electrophoresis on a 1% agarose gel to monitor the degradation and contamination levels. The extracted RNA was then subjected to monitor the degradation, contamination levels, purity, and quantity of RNA.

Transcriptomic analyses of subpetiolar buds from different tissues, developmental stages, and strains were performed with three biological replicates for NGS library preparation. After the RNA samples passed a quality assessment, mRNA was first isolated and purified, followed by reverse transcription to synthesize cDNA. The library preparation involved several steps, including end repair, A-tailing, adapter ligation, purification, PCR amplification, denaturation, and circularization, using the NadPrep^®^ DNA universal library construction kit (Nanodigmbio, Nanjing, China). Sequencing was conducted using the MGI-SEQ 2000 platform (MGI Tech Co., Ltd., Shenzhen, China).

For the Nanopore full-length transcriptomic analysis, qualified RNA samples without a replicate were subjected to reverse transcription with connected primers to amplify mRNA. AMPure magnetic beads were employed for purification, and the purified cDNA was ligated with sequencing adapters from the SQK-PCS109 kit (Beckman Coulter, Brea, CA, USA). The quantification of the constructed cDNA library was performed using Qubit, and after passing the quality assessment, sequencing was conducted on the PromethION platform (Oxford Nanopore Technologies, Oxford, UK).

The data of the transcriptome used in this project was deposited into CNGBdb under the accession code CNP0004600.

### 4.4. Assembling Contigs from NGS Reads

Low-quality reads in the NGS data were filtered out (the base with quality values below Q15 greater than 20% and reads length shorter than 30 bp) using Fastp [87] with the following parameters: –u = 20 –l = 30. The contigs of transcripts that consist of diploids and tetraploids were assembled using Trinity [88] with parameters “–edge-thr  =  0, –flow-thr  =  0”.

### 4.5. Collection of Full-Length Corrected Transcripts

For clean reads from the ONT sequencing, full-length reads were identified and primers were trimmed using Pychopper (github.com/epi2me-labs/pychopper, accessed on 12 December 2022) with default parameters and the PCS109 primer hmms. Nanofilt [89] was then applied for quality control with the parameters “-q 7 -l 200 --headcrop 50 --tailcrop 50”. The first and last 50 bp of all reads were removed, as well as all reads with a minimum average read quality score of less than 7 and those with a length of less than 200 bp. LORDEC [90] was utilized to correct the full-length transcriptome using NGS reads. CD-HIT [91] was then employed to cluster the full-length transcripts to align shorter sequences to longer ones and remove redundant sequences with an identity greater than 99%.

### 4.6. Mapping Finding between Full-Length Reads and Contigs

The approach we used to map and identify the longest contig datasets was based on the principles of hybrid sequencing and map finding (HySeMaFi) [38], and the basic principle of the analysis is outlined in Figure S1. Briefly, the long reads obtained from the ONT sequencing captured full-length transcripts representing the actual transcribed isoforms. The contigs were assembled based on NGS reads that represented both true and false isoforms containing all possible exons. The blat was used to map between the isoforms and the longest contig data sets with the following parameters: minScore = 200, maxGap = 2, and minIdentity = 99 [92]. Subsequently, the longest mapped contigs were selected to constitute the reference library covering all aligned isoforms with high similarity. 

### 4.7. Differential Gene Expression Analysis and Isoform Comparison between Hexaploids and Dodecaploids

NGS reads were aligned with the longest mapped contigs as a reference transcriptome collection using Bowtie2 [93]. Subsequently, RSEM [94] was employed to normalize RNA-seq fragment counts to fragments per kilobase per million reads (FPKM) and construct the transcriptome expression matrix. Differential expression analysis was performed using DESeq2 [95], where genes with an expression fold change greater than 2 and a *p*-value ≤ 0.05 were selected as differentially expressed genes. The expression heatmaps were generated using the R package Pheatmap [96].

The results of map finding between isoforms and contigs were converted to gff3 format using a custom script, and gffcompare [97] was used to compare isoform structural differences between the hexaploids and dodecaploids.

### 4.8. Alternative Splicing Analysis

Alternative splicing analysis based on isoforms and contig structures was performed using SUPPA2 [98]. NGS reads were aligned to isoforms using Bowtie2 [93], and then Salmon [99] was used for quantification based on the alignment results. SUPPA2 was also used to perform differential analysis of selective splicing based on the quantification results of the NGS reads. Here, ploidy-responsive AS events were defined by the criteria “false discovery rate < 0.05” and ≥20% variation in PSI (event PSI difference (|ΔPSI|)), and the plot_isoform_usage script was used for visualization [98].

### 4.9. qRT-PCR Validation of AS Events

Complementary DNA (cDNA) was synthesized using a HiScript III 1st Strand cDNA Synthesis Kit (+gDNA wiper; Vazyme, Nanjing, China). Quantitative real-time PCR (qRT-PCR) was performed using cDNA as a template and using ChamQ SYBR Color qPCR Master Mix (Low ROX Premixed; Vazyme, Nanjing, China) in an Applied Biosystems 7500 Fast Real-Time PCR System (Applied Biosystems, Waltham, MA, USA) and following the manufacturer’s instructions. The qRT-PCR amplifications were performed in a 10 μL volume reaction containing a 1.0 μL template, 5.0 μL ChamQ SYBR Color qPCR Master Mix, 0.2 μL forward and reverse primers (10 μL mol/μL), and 3.6 μL water. The qRT-PCR reaction program was 95 °C for 30 s, 95 °C for 10 s, and 60 °C for 30 s (40 cycles). Primers for qRT-PCR were designed using Primer Premier 5 (Lalitha, 2000). The *PaTPI* (*P*. × *acerifolia* triose phosphate isomerase) was used as reference genes to normalize all data referred to in previous studies [100]. The expression levels of a relative gene were detected using the 2^−∆∆CT^ method. Data were presented as the mean value ± SD (standard deviation) from three biological replicates. The histograms were drawn using GraphPad Prism 9 (graphpad.com). The primers used are shown in Supplementary Table S20.

## 5. Conclusions

The dodecaploid *P.* × *acerifolia* has fewer flowers compared with its hexaploid counterparts. Full-length transcriptome analysis revealed that the novel transcriptional events specific to the dodecaploids contributed to repairing the damage of genomic rearrangements and chromosome mismatches resulting from polyploidization. In addition, several genes related to flowering transition and flower development showed significant differential expression in the dodecaploids compared with the hexaploids. Analysis of alternative splicing indicated that the dodecaploids generated a greater number of splice variants than the hexaploids in *P.* × *acerifolia*. The integration of transcriptomic and RT-qPCR analyses revealed differential alternative splicing of certain flowering transition and flower development genes in dodecaploid *P.* × *acerifolia* compared with the hexaploid. Taken together, our results provide new insights into the transcriptional and alternative splicing regulatory patterns and molecular mechanisms underlying the reduced flowering phenotype in polyploidized *P.* × *acerifolia*. The knowledge gained from this study will contribute to the creation of polyploid *Platanus* germplasms with few flowers.

## Data Availability

The data generated during the current study are available in the China National GeneBank DataBase (CNGBdb) repository with the project accession code CNP0004600.

## Supplementary Materials

The following supporting information can be downloaded from https://www.mdpi.com/article/10.3390/ijms241914486/s1.
