# Supplementary material for: The Abundant and Unique Transcripts and Alternative Splicing of the Artificially Autododecaploid London Plane (Platanus × acerifolia)
Source: Int J Mol Sci. 2023 Sep 23;24(19):14486. doi: 10.3390/ijms241914486 (PMC10572260; doi:10.3390/ijms241914486)
Supplement: Supplementary file 1 [file ijms-24-14486-s001.zip › Figure-Supplementary.pdf]

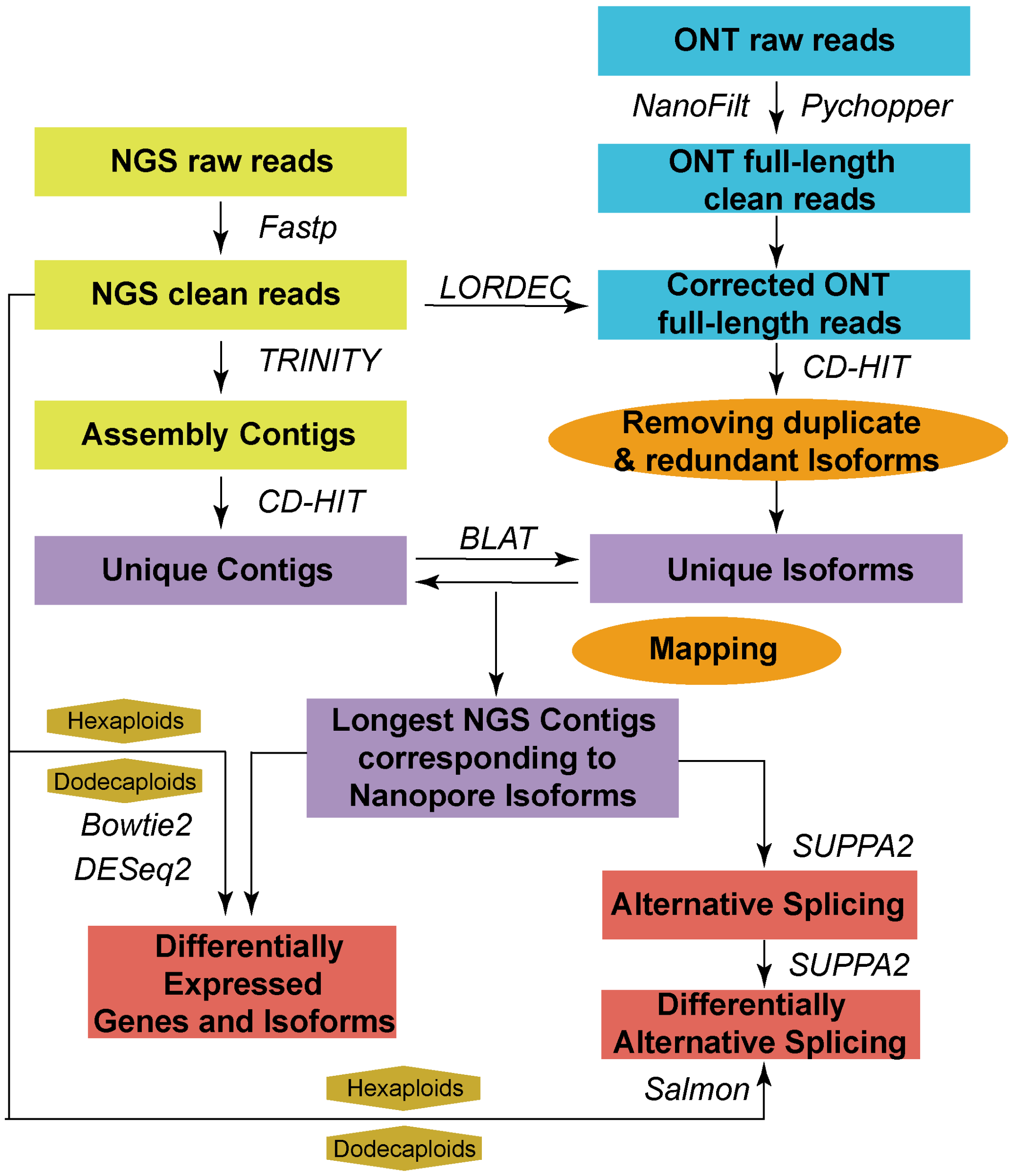

Figure S1. Strategy to decipher the splicing and expression in the transcriptome of *Platanus*.

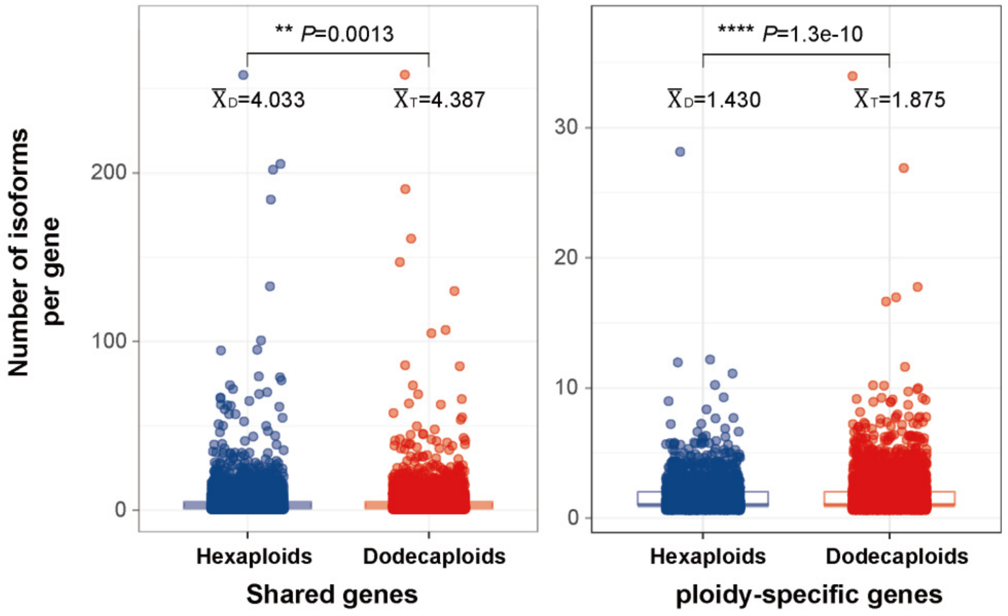

Figure S2. Number of isoforms per shared gene and ploidy-specific in hexaploids and dodecaploids.

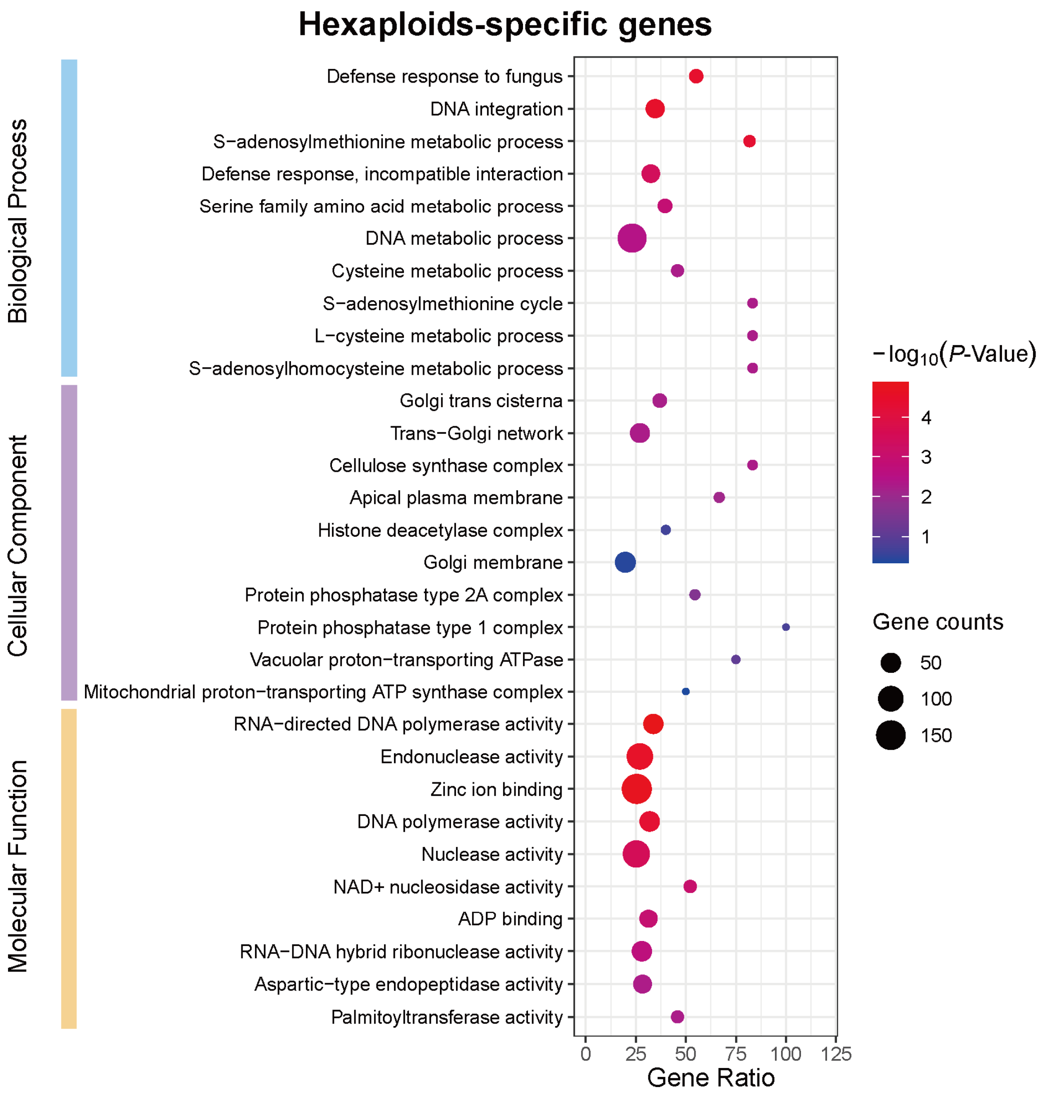

Figure S3. The GO terms enrichment analysis of genes specifically transcripted in hexaploids.

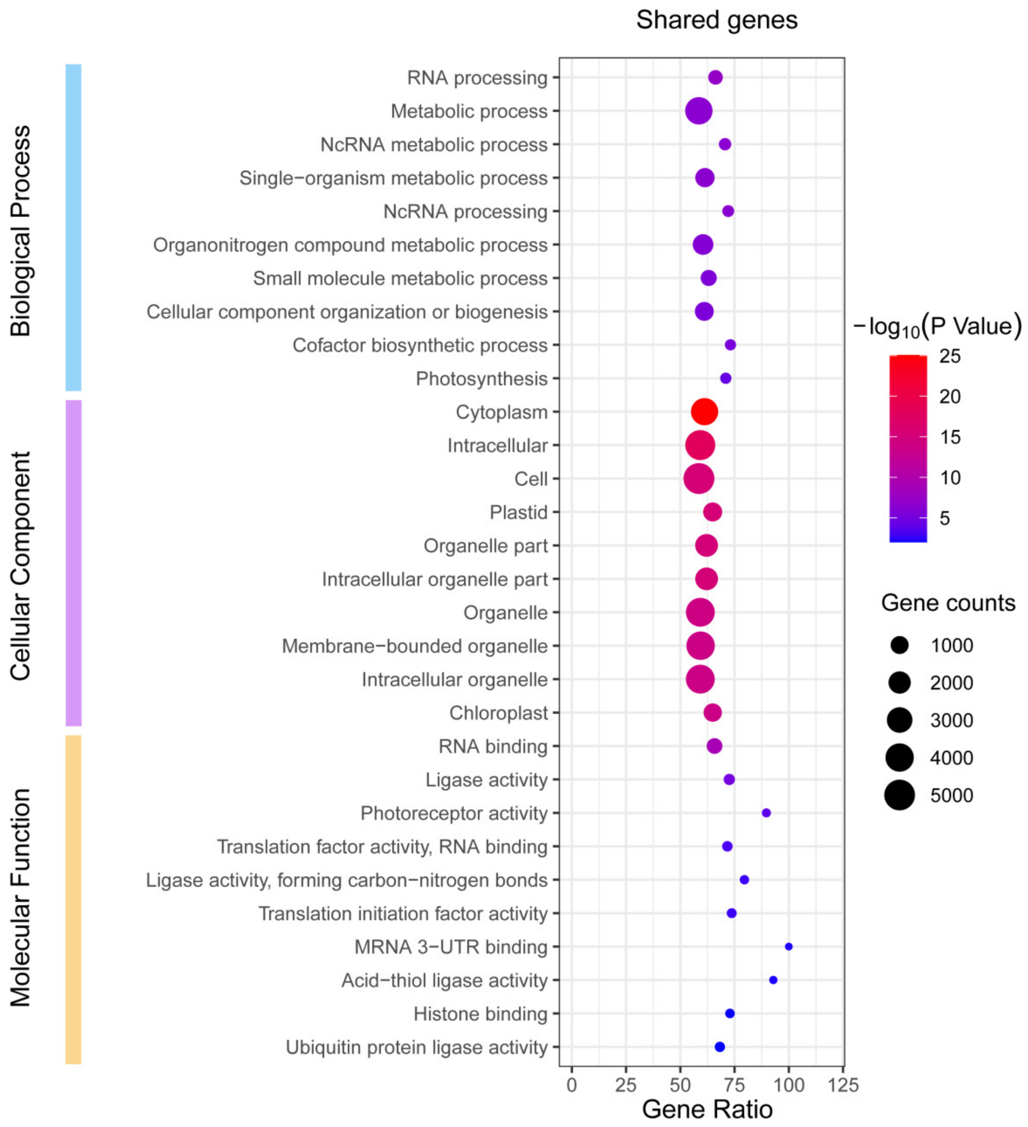

Figure S4. The GO terms enrichment analysis of genes transcripted in both hexaploids and dodecaploids.

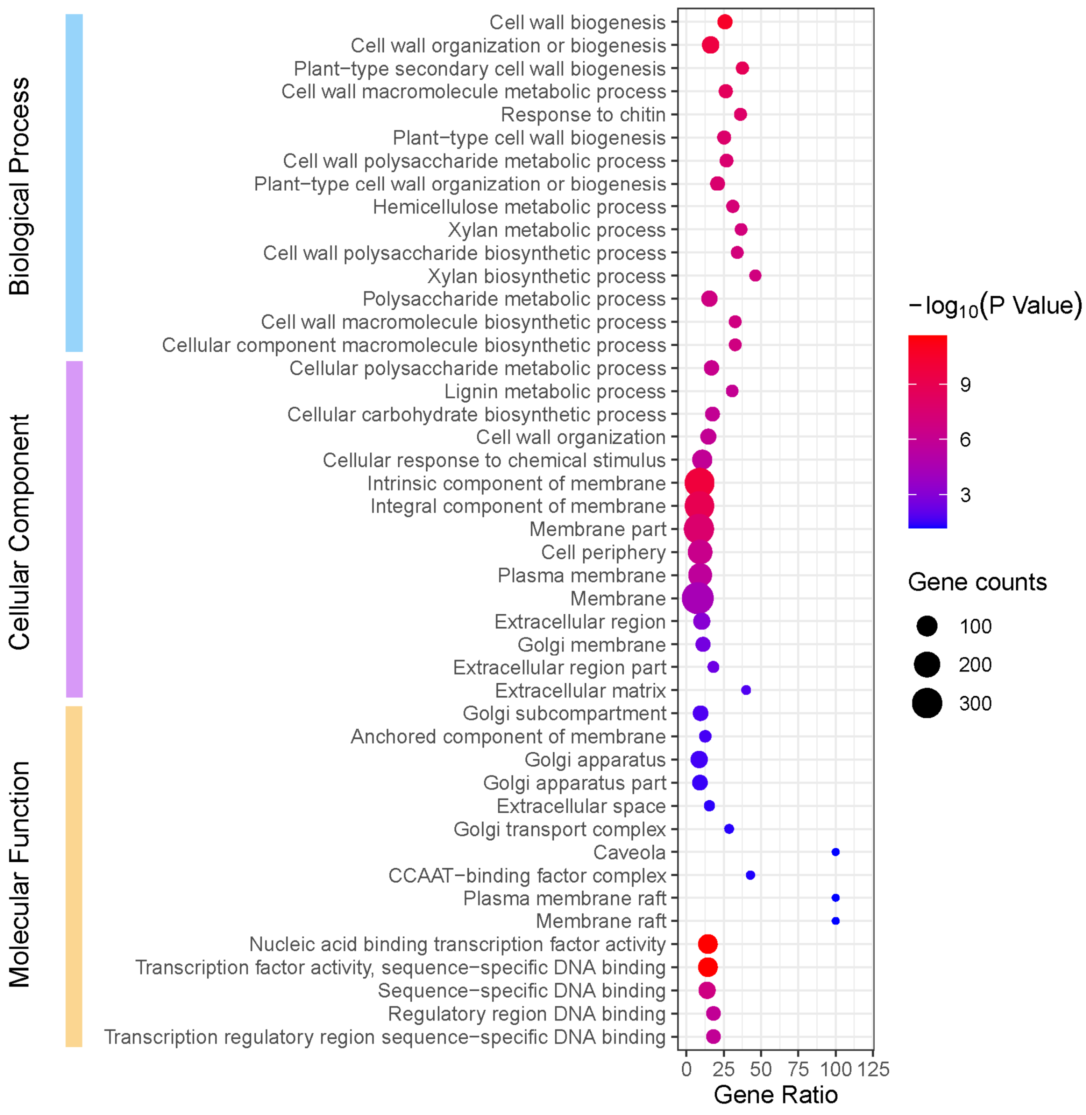

Figure S5. The GO terms enrichment analysis of up-regulated DEGs in dodecaploids at Apr. 21st.

.

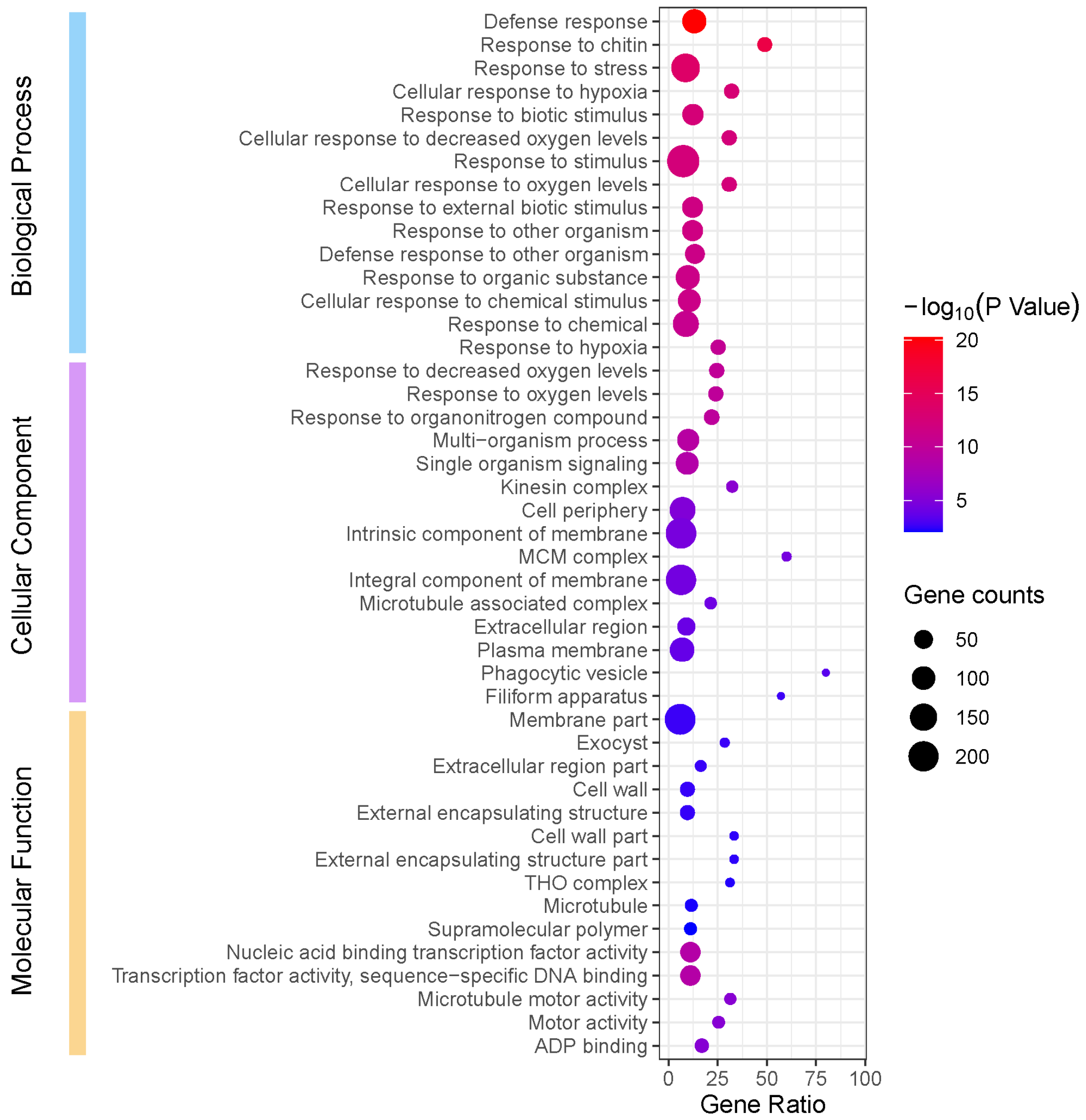

Figure S6. The GO terms enrichment analysis of up-regulated DEGs in dodecaploids at May. 05th.

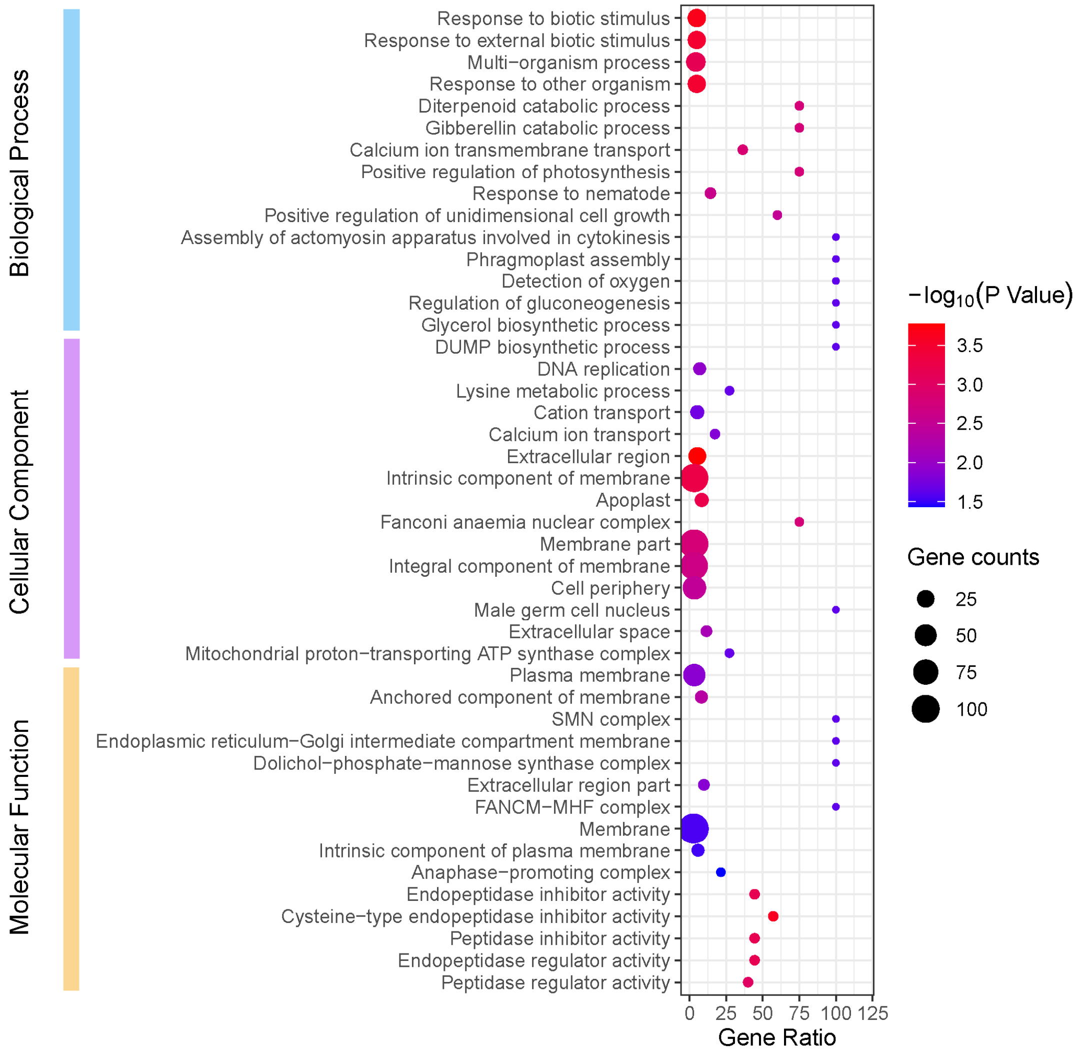

Figure S7. The GO terms enrichment analysis of up-regulated DEGs in dodecaploids at May. 20th.

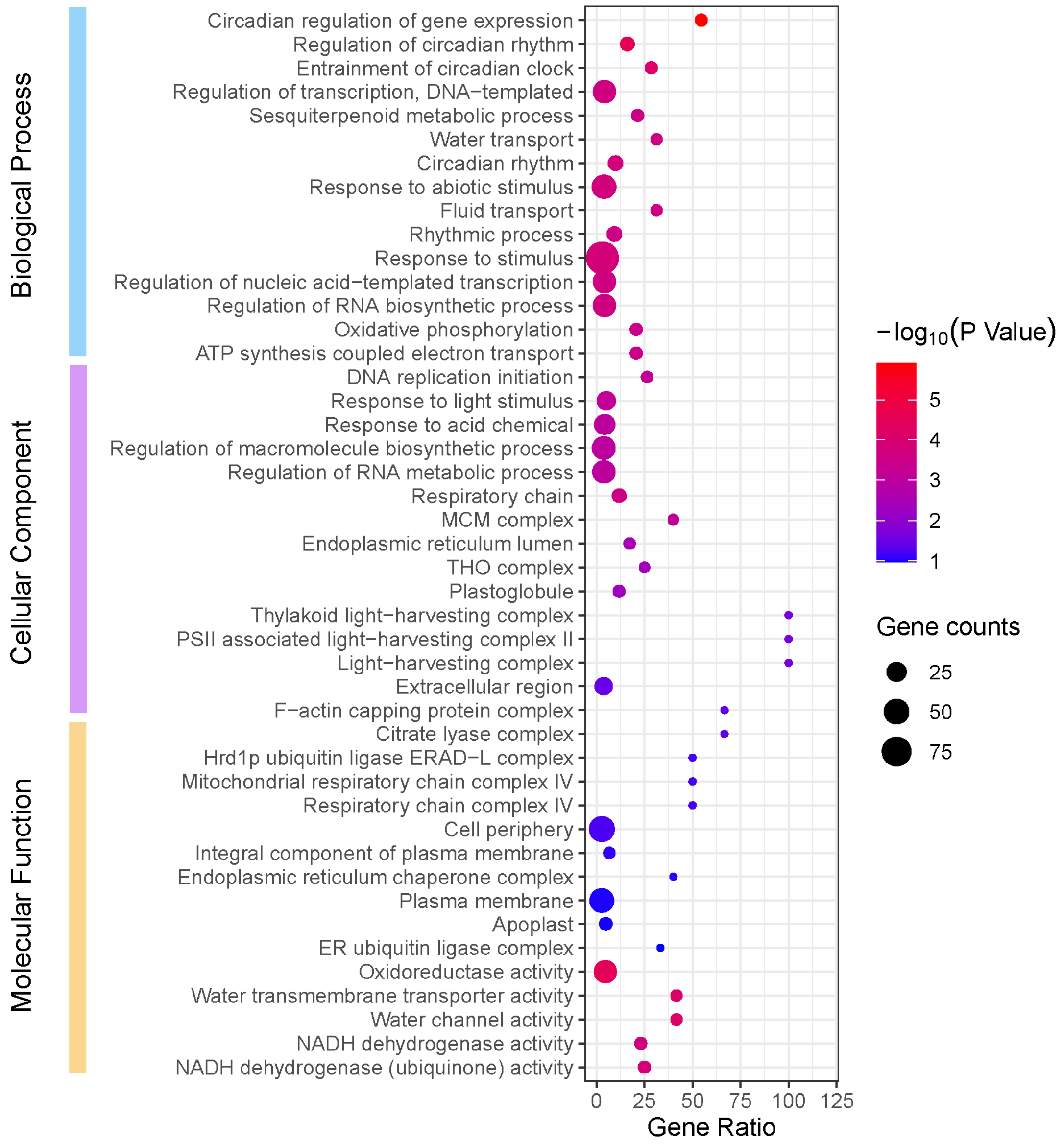

Figure S8. The GO terms enrichment analysis of up-regulated DEGs in dodecaploids at Jun. 05th.

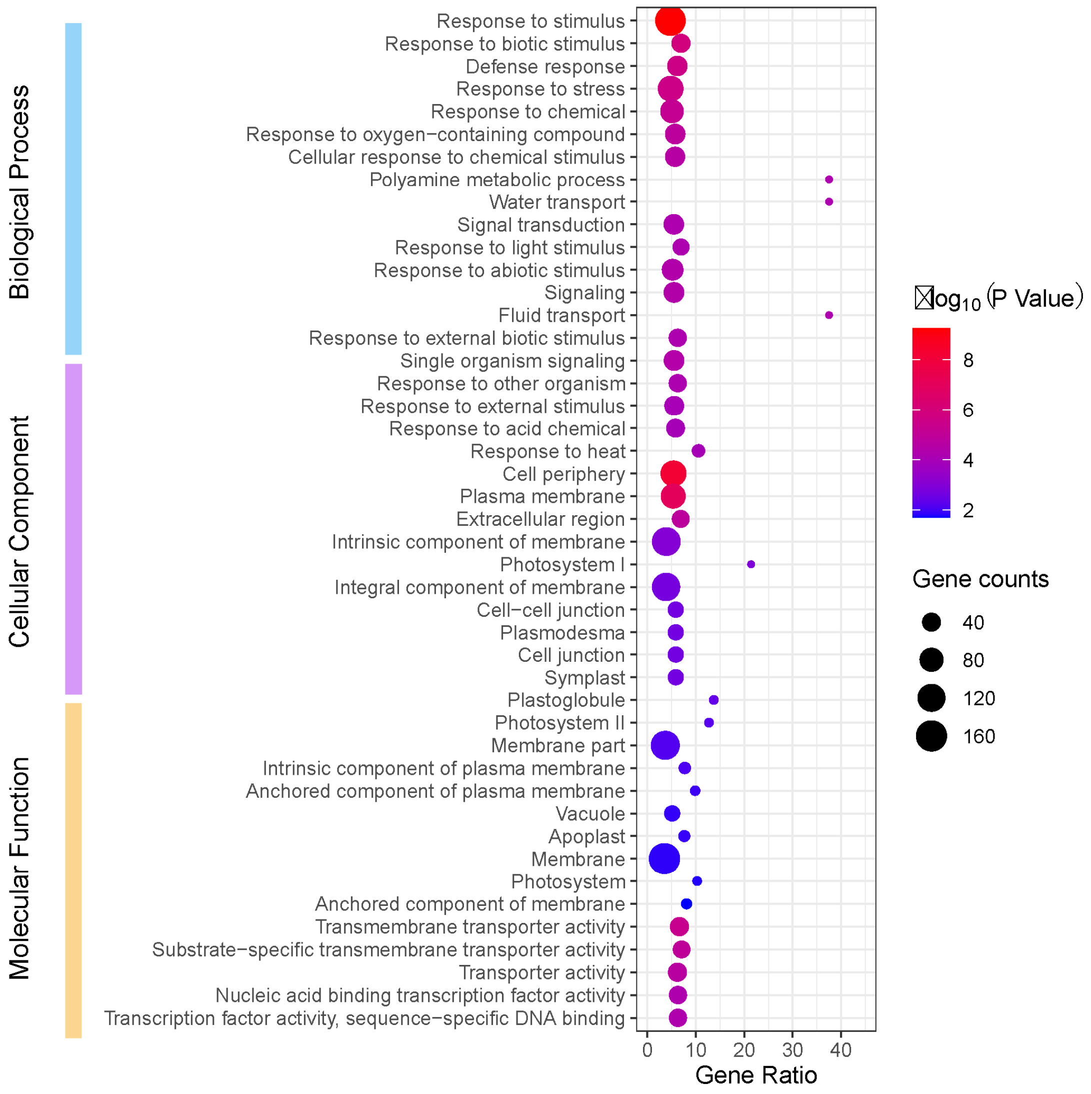

Figure S9. The GO terms enrichment analysis of up-regulated DEGs in dodecaploids at Jun. 23rd.

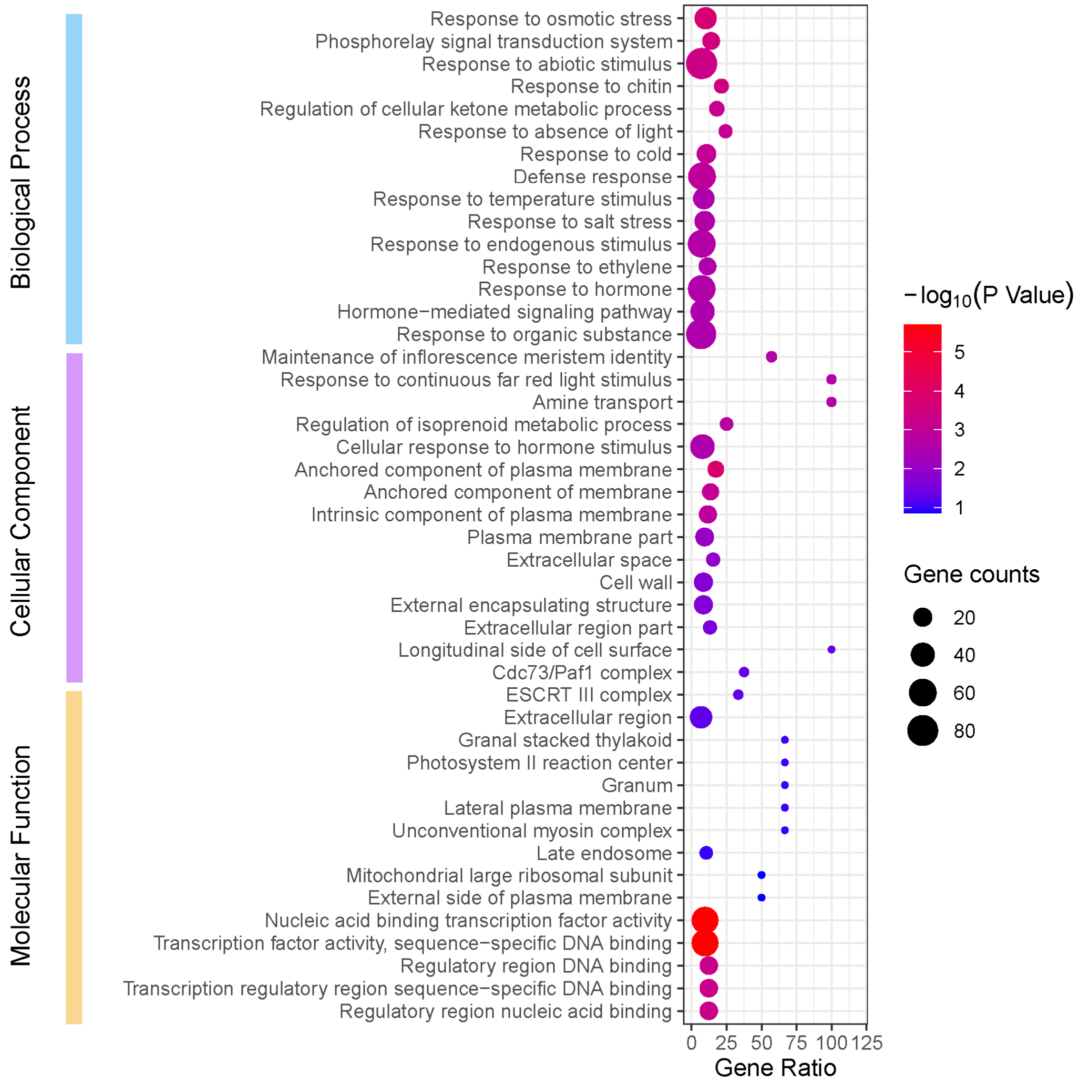

Figure S10. The GO terms enrichment analysis of up-regulated DEGs in dodecaploids at Jul. 20th.

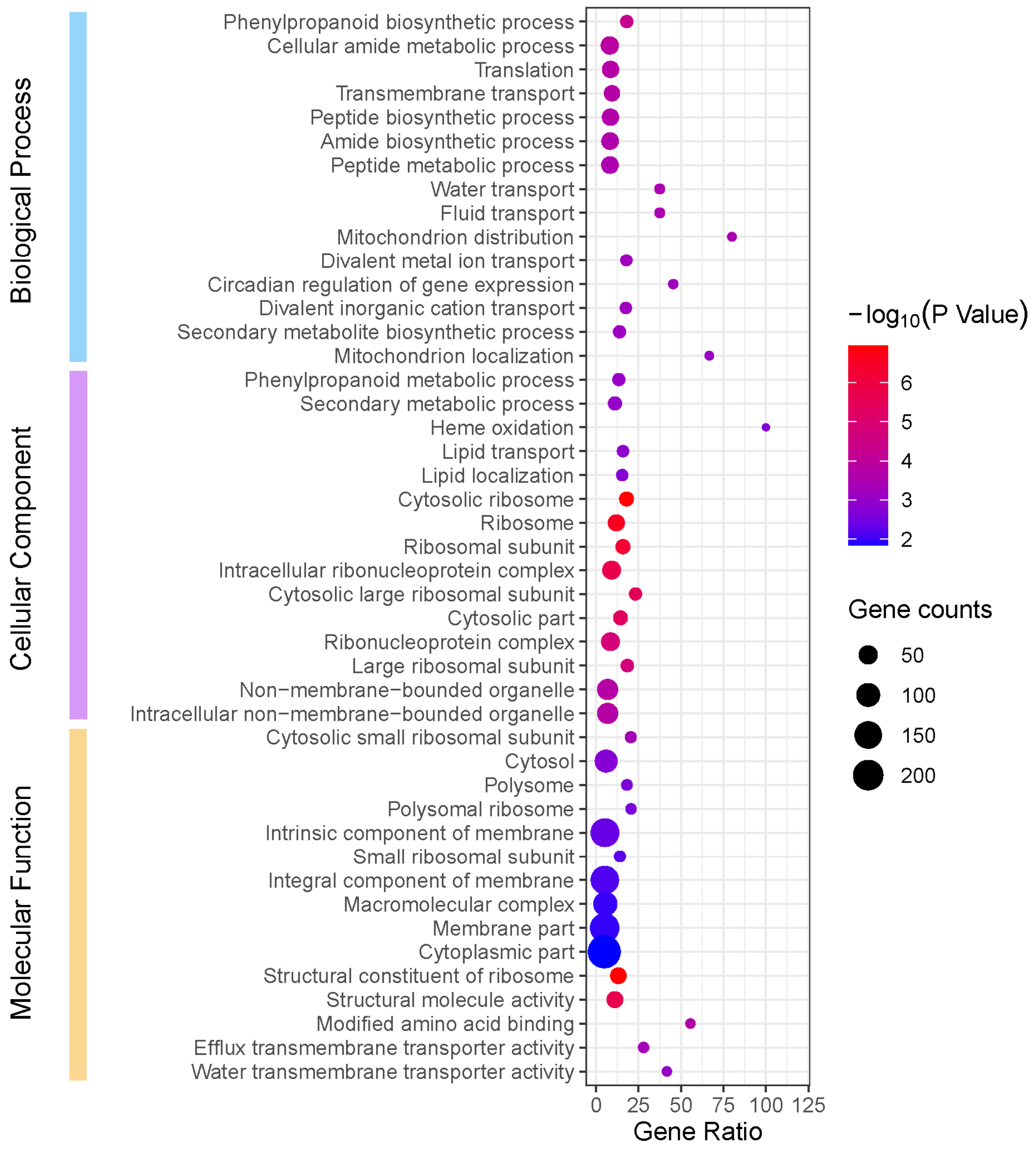

Figure S11. The GO terms enrichment analysis of down-regulated DEGs in dodecaploids at Apr. 21st.

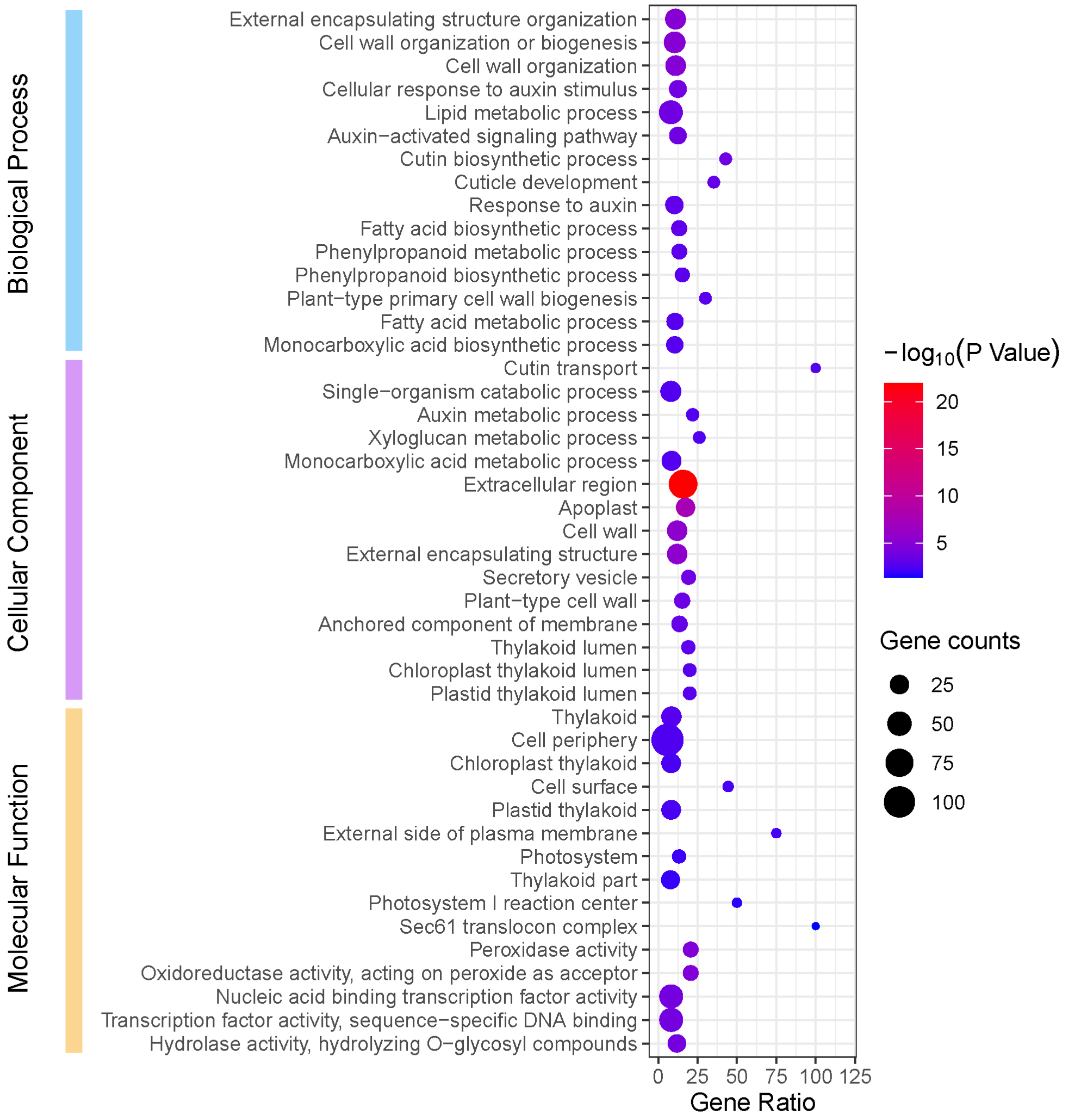

Figure S12. The GO terms enrichment analysis of down-regulated DEGs in dodecaploids at May. 05th.

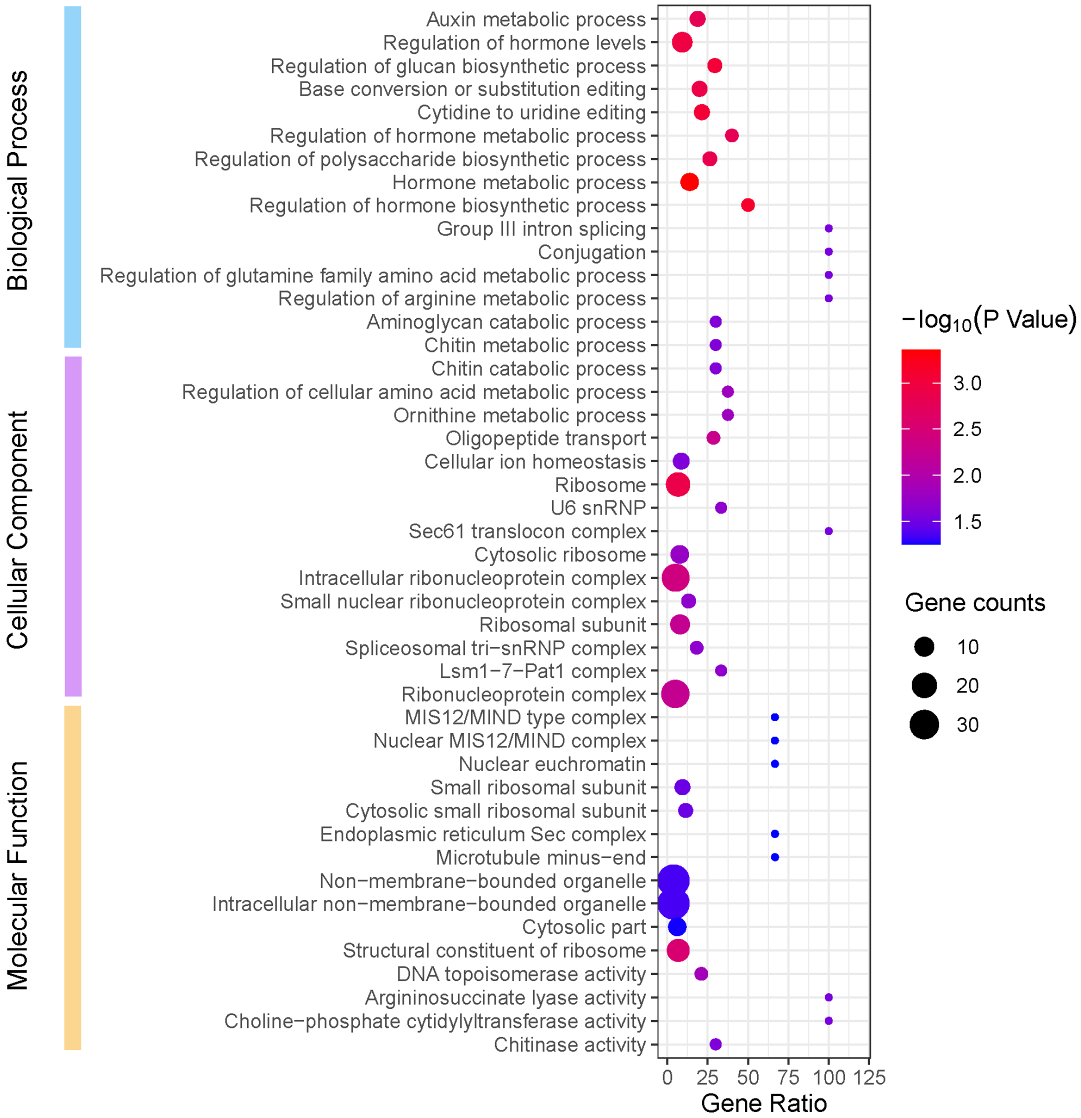

Figure S13. The GO terms enrichment analysis of down-regulated DEGs in dodecaploids at May. 20th.

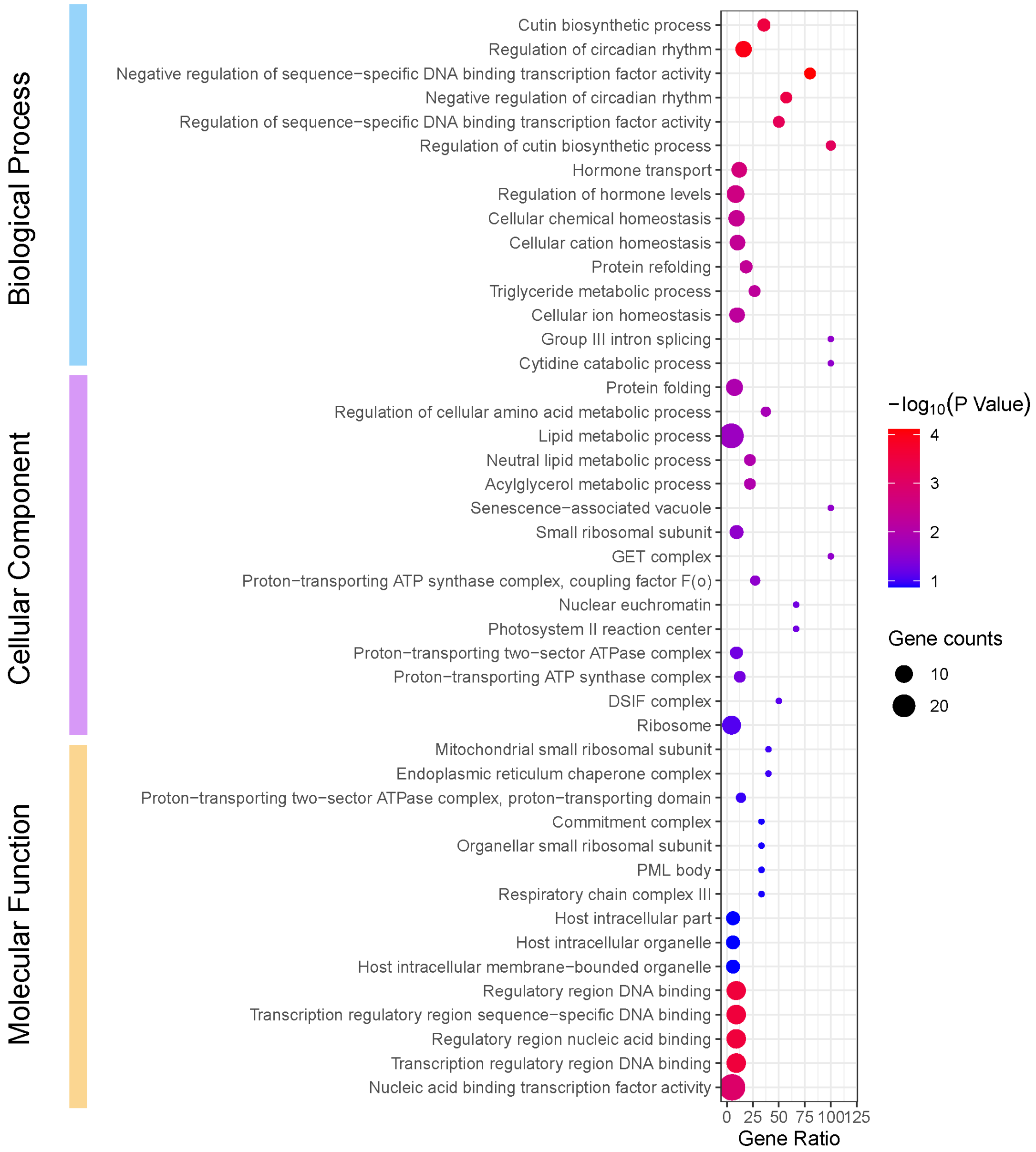

Figure S14. The GO terms enrichment analysis of down-regulated DEGs in dodecaploids at Jun. 05th.

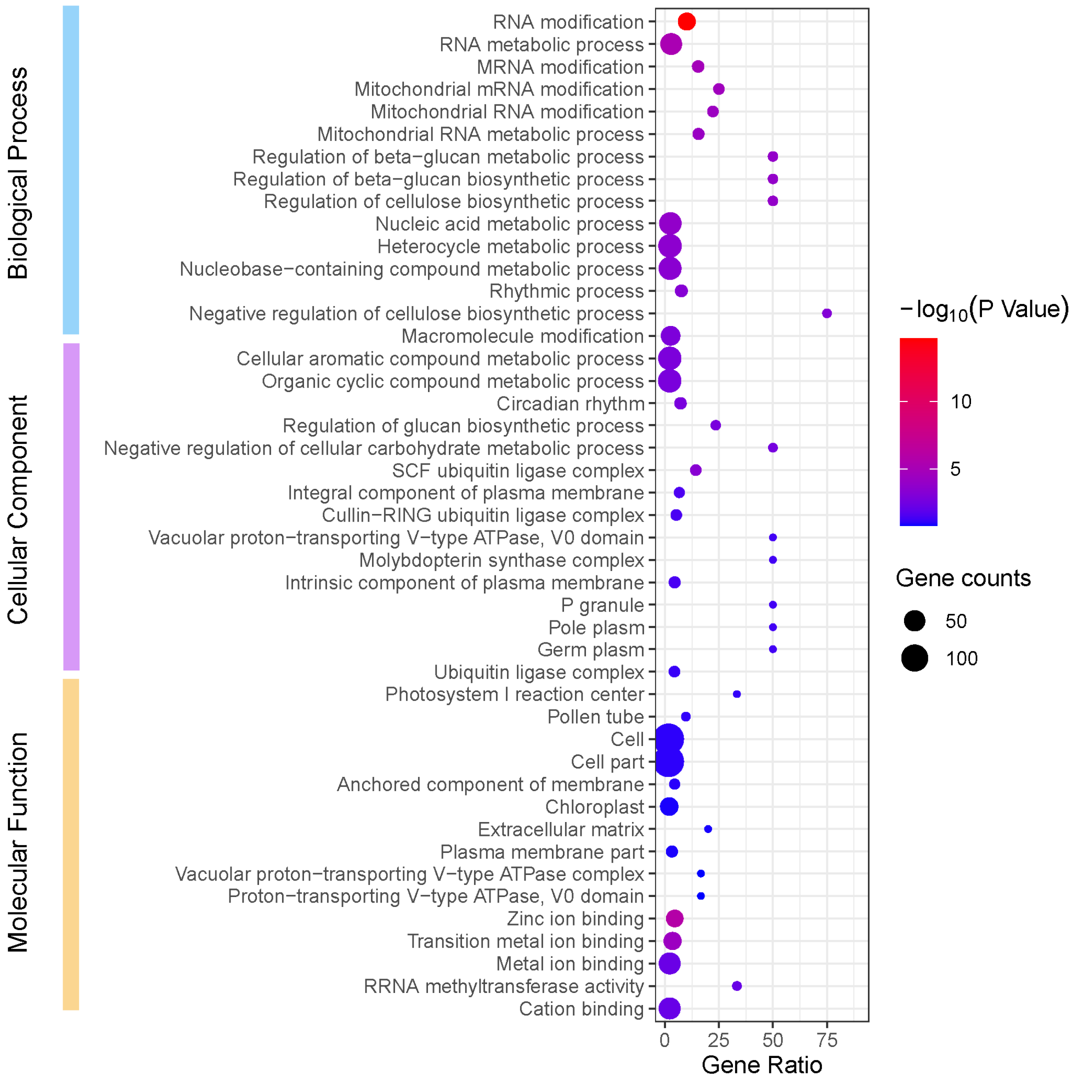

Figure S15. The GO terms enrichment analysis of down-regulated DEGs in dodecaploids at Jun. 23rd.

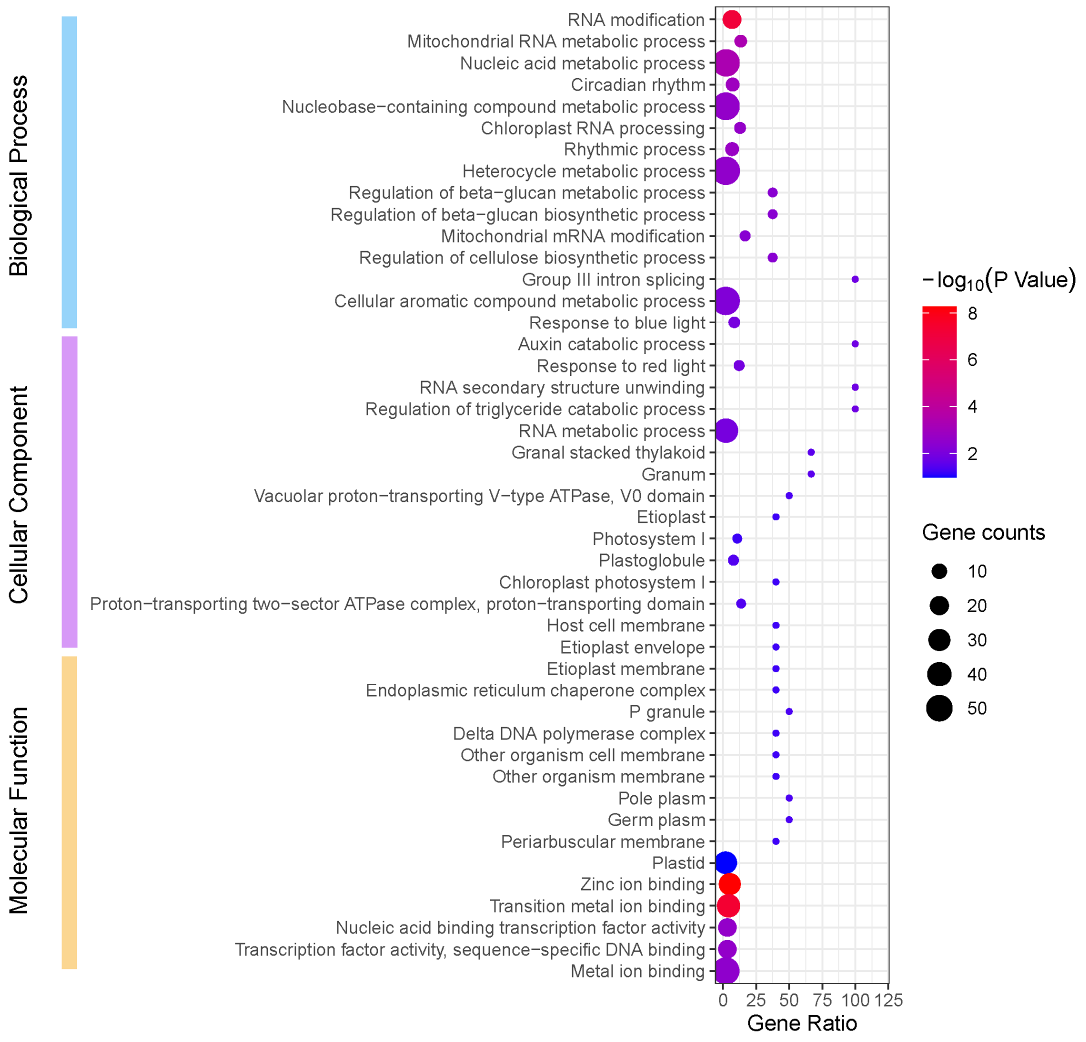

Figure S16. The GO terms enrichment analysis of down-regulated DEGs in dodecaploids at Jul. 20th.

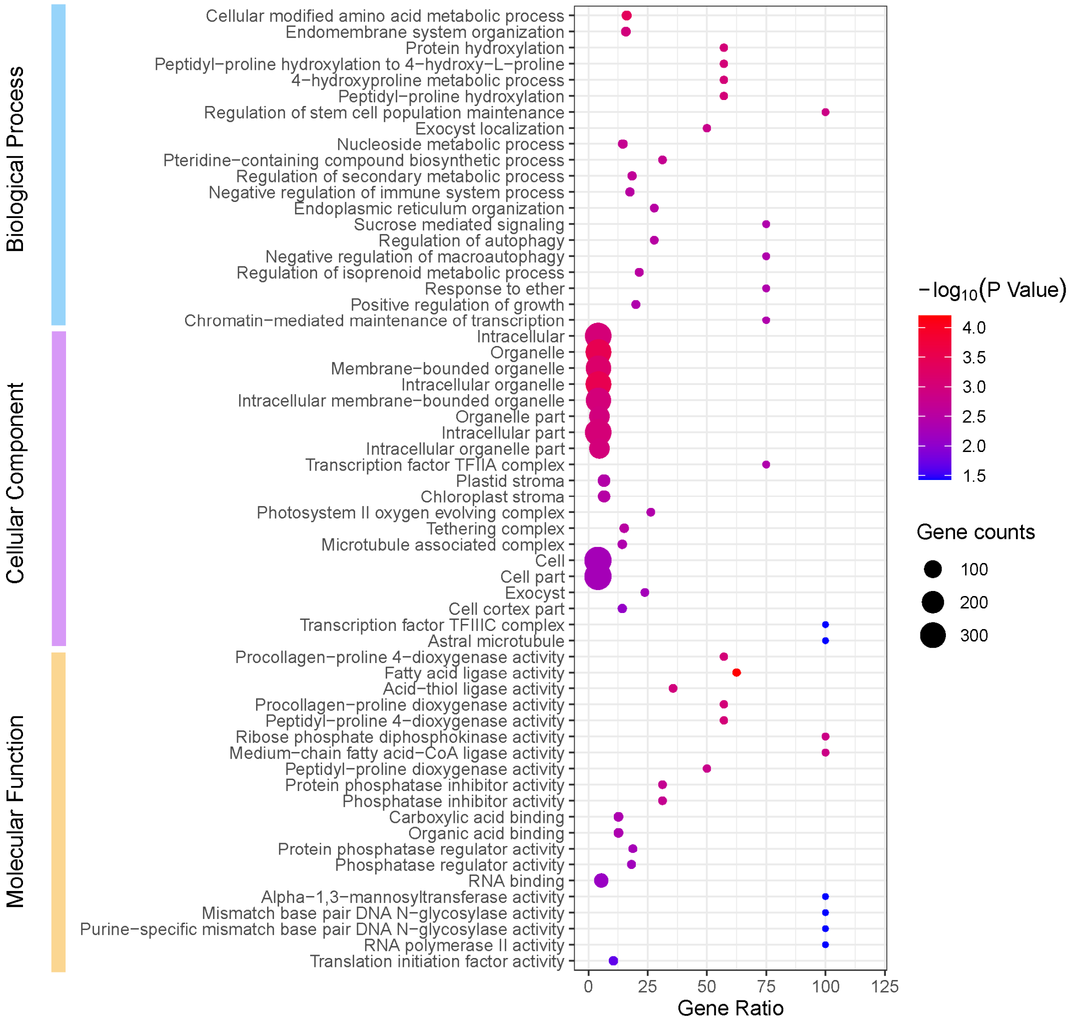

Figure S17. The GO terms enrichment analysis of DSGs in Apr. 21st.

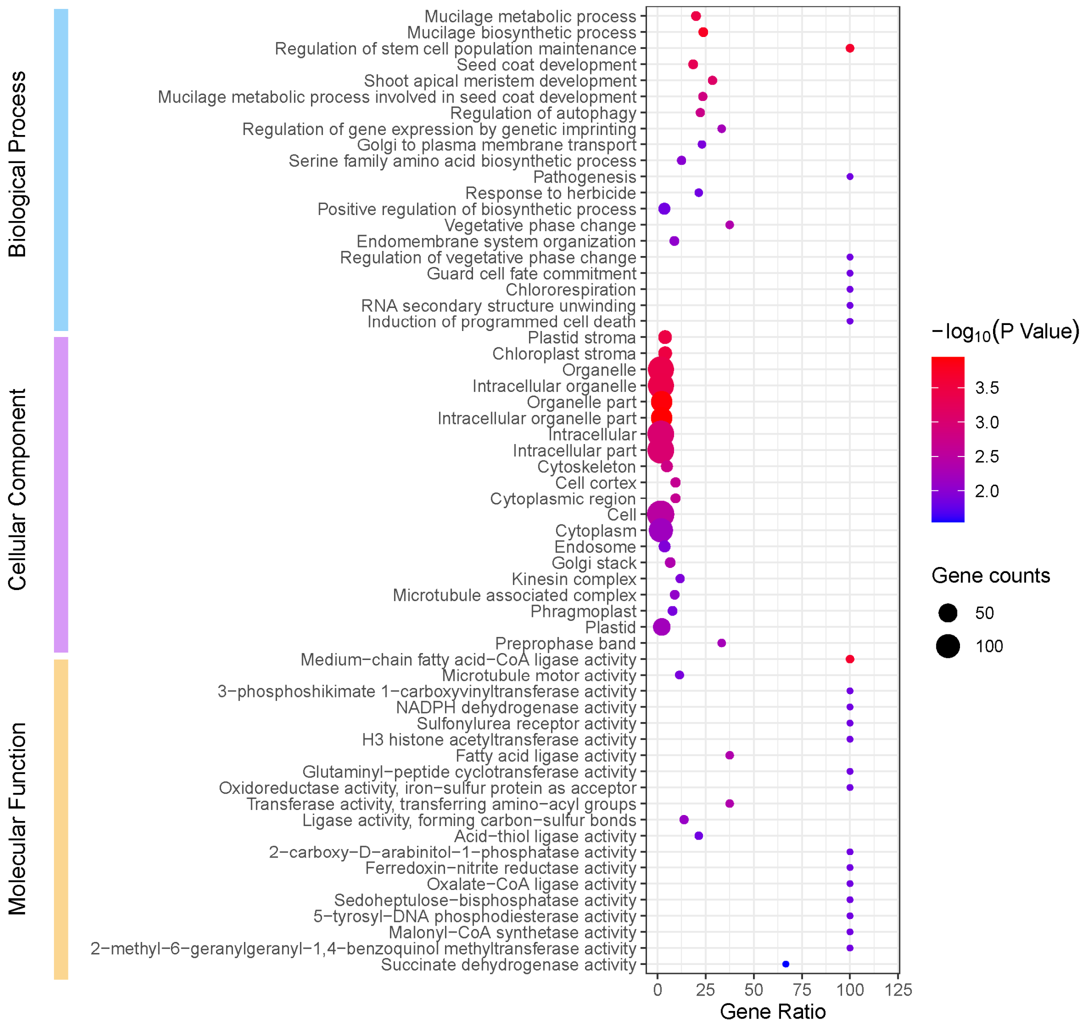

Figure S18. The GO terms enrichment analysis of DSGs in May. 05th.

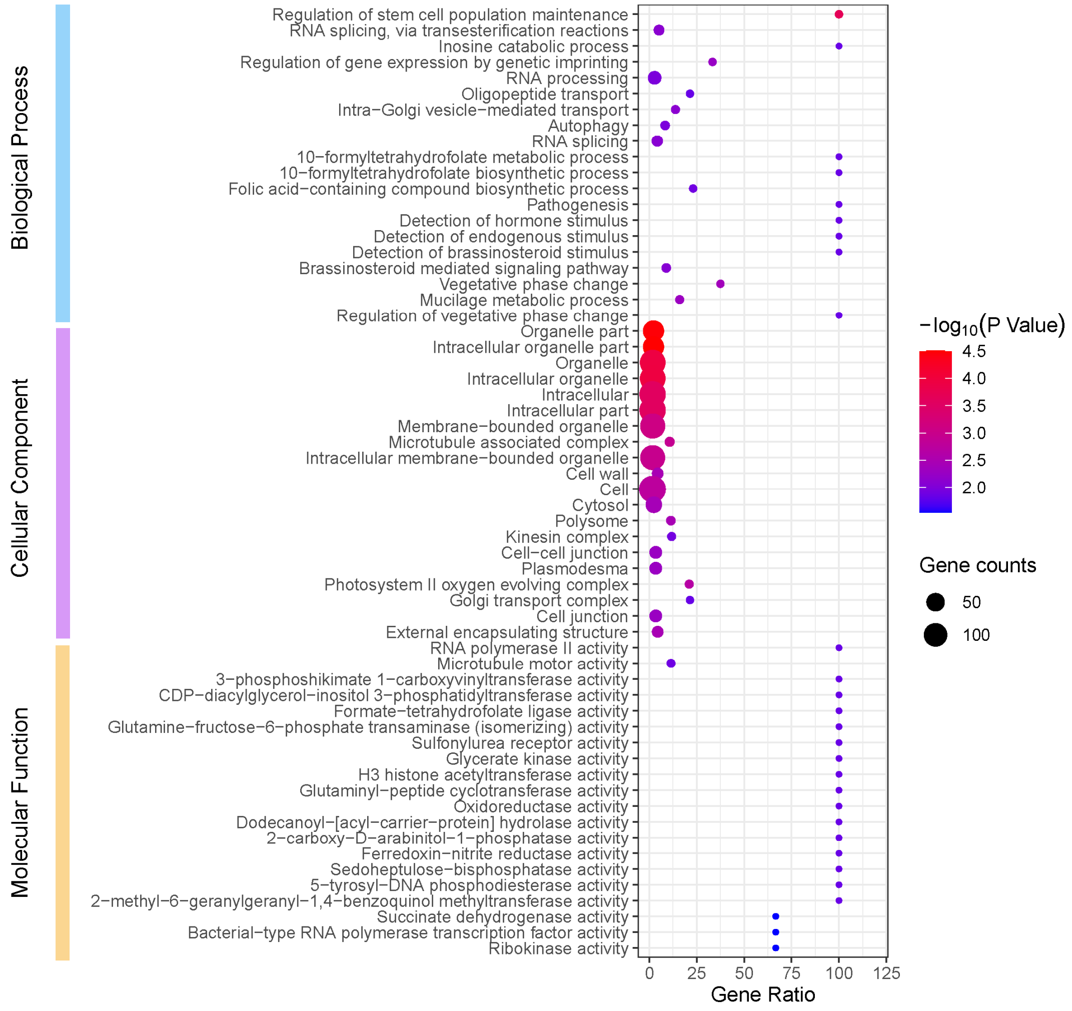

Figure S19. The GO terms enrichment analysis of DSGs in May. 20th.

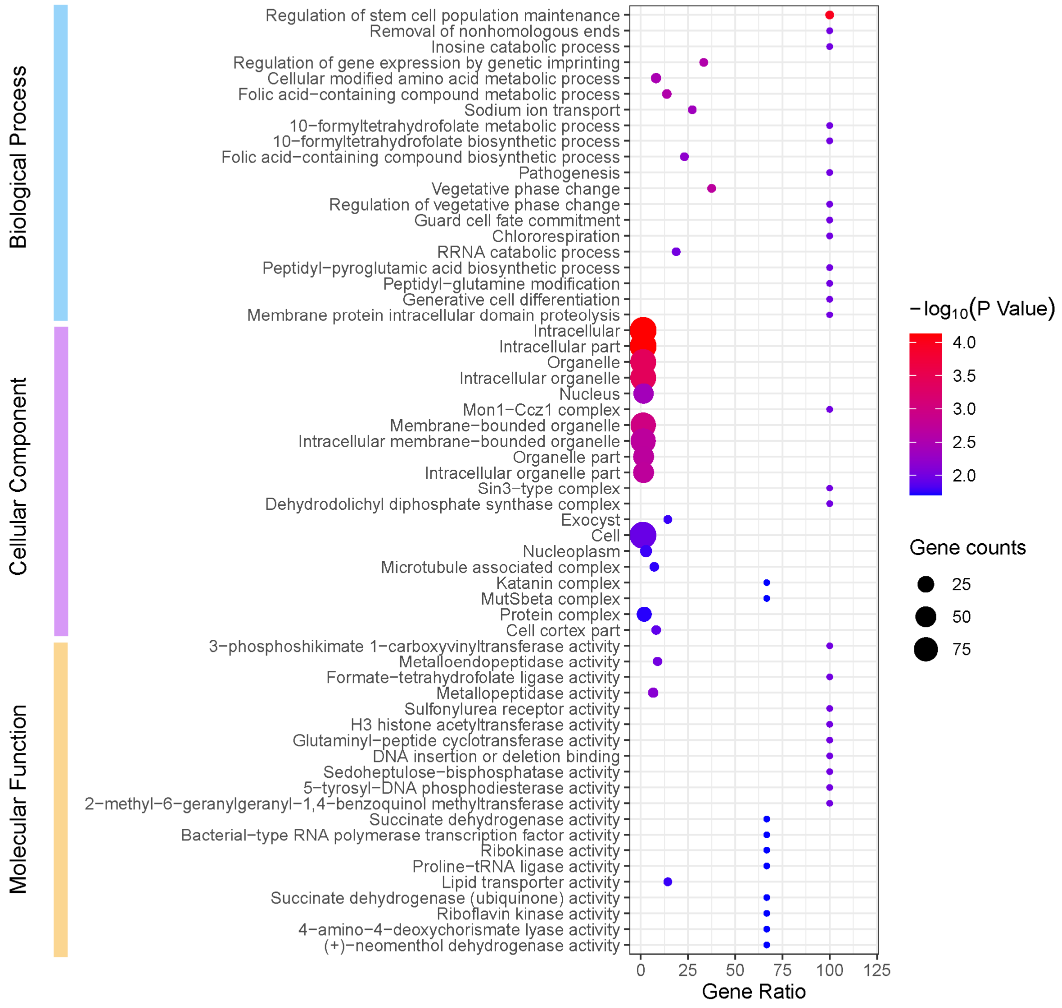

Figure S20. The GO terms enrichment analysis of DSGs in Jun. 05th.

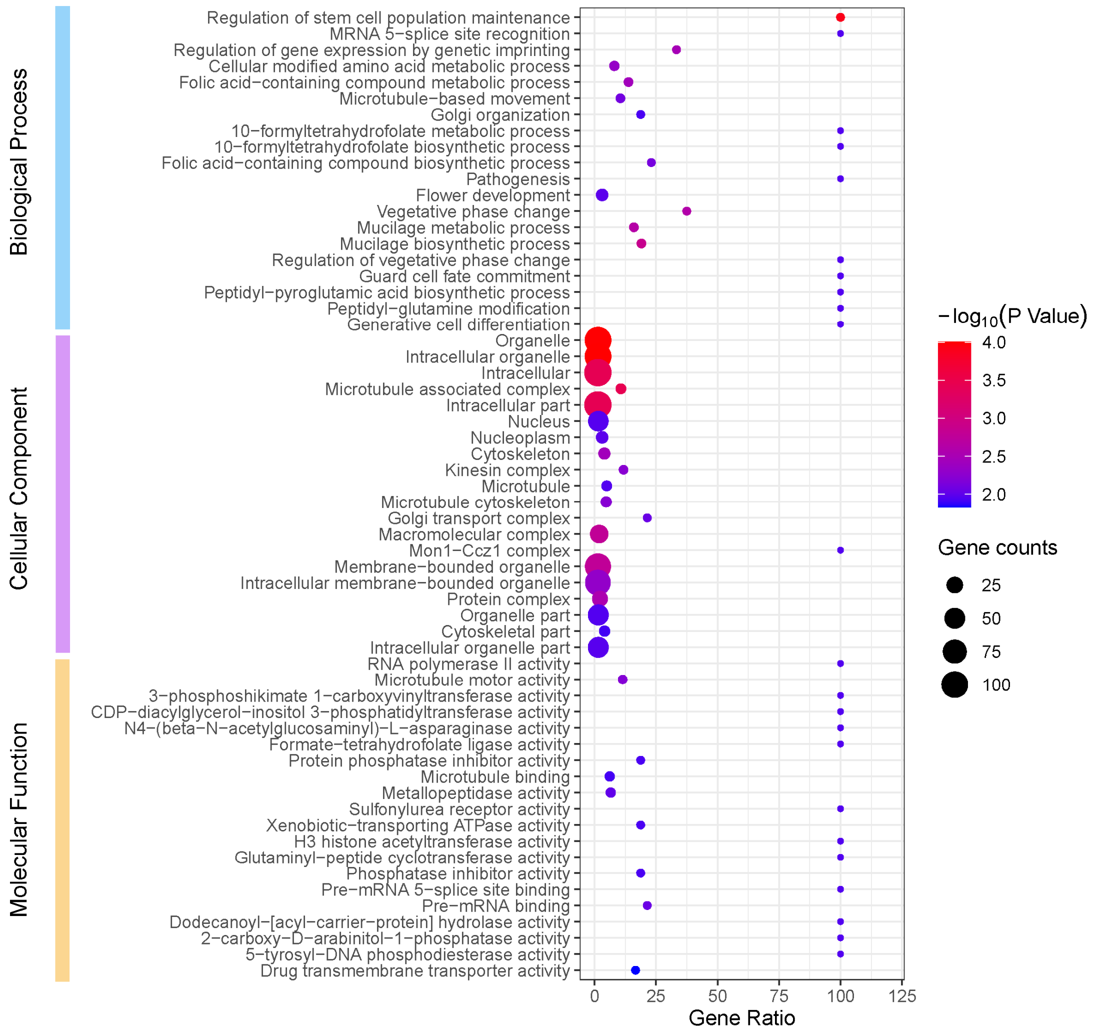

Figure S21. The GO terms enrichment analysis of DSGs in Jun. 23rd.

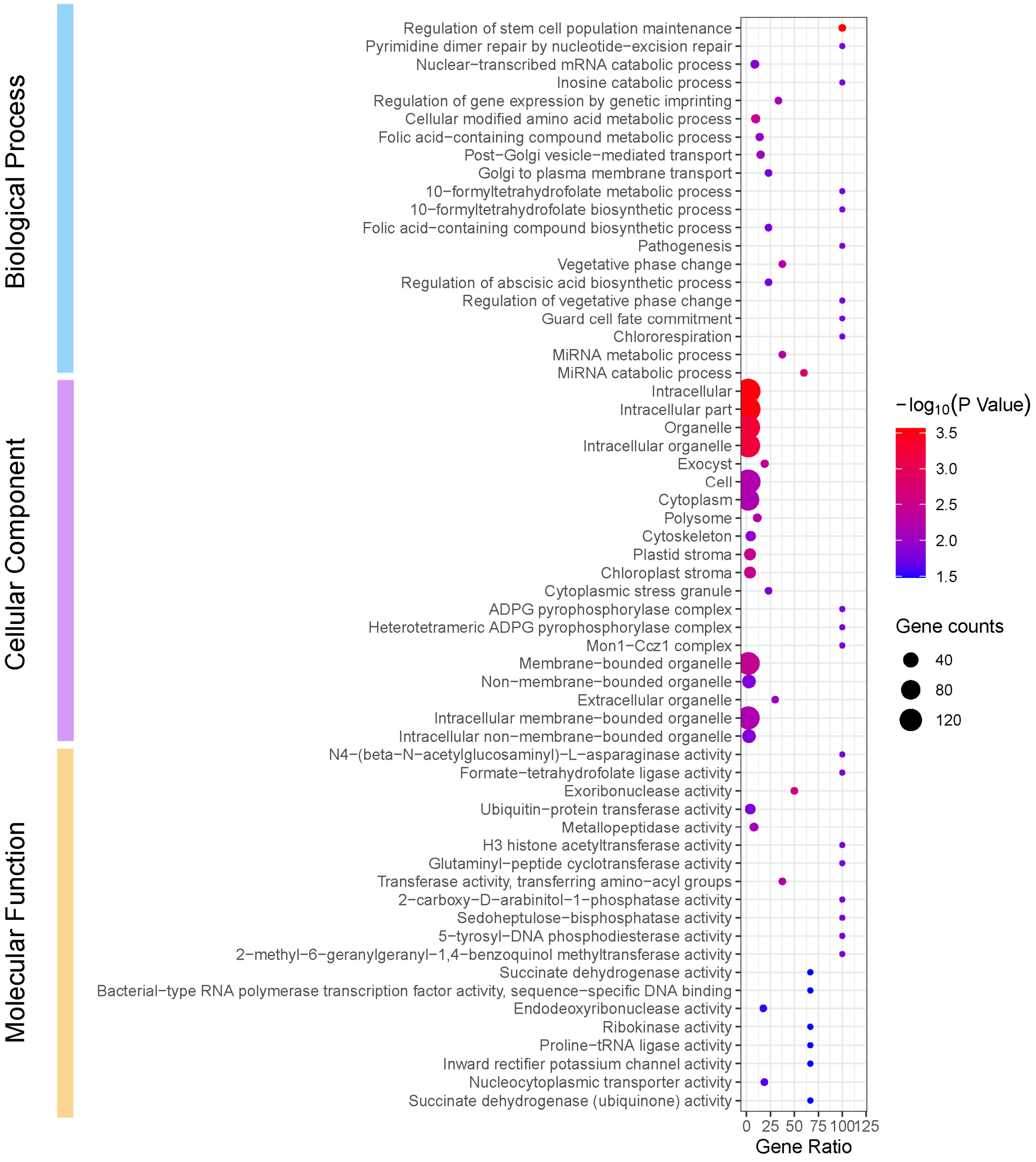

Figure S22. The GO terms enrichment analysis of DSGs in Jul. 20th.

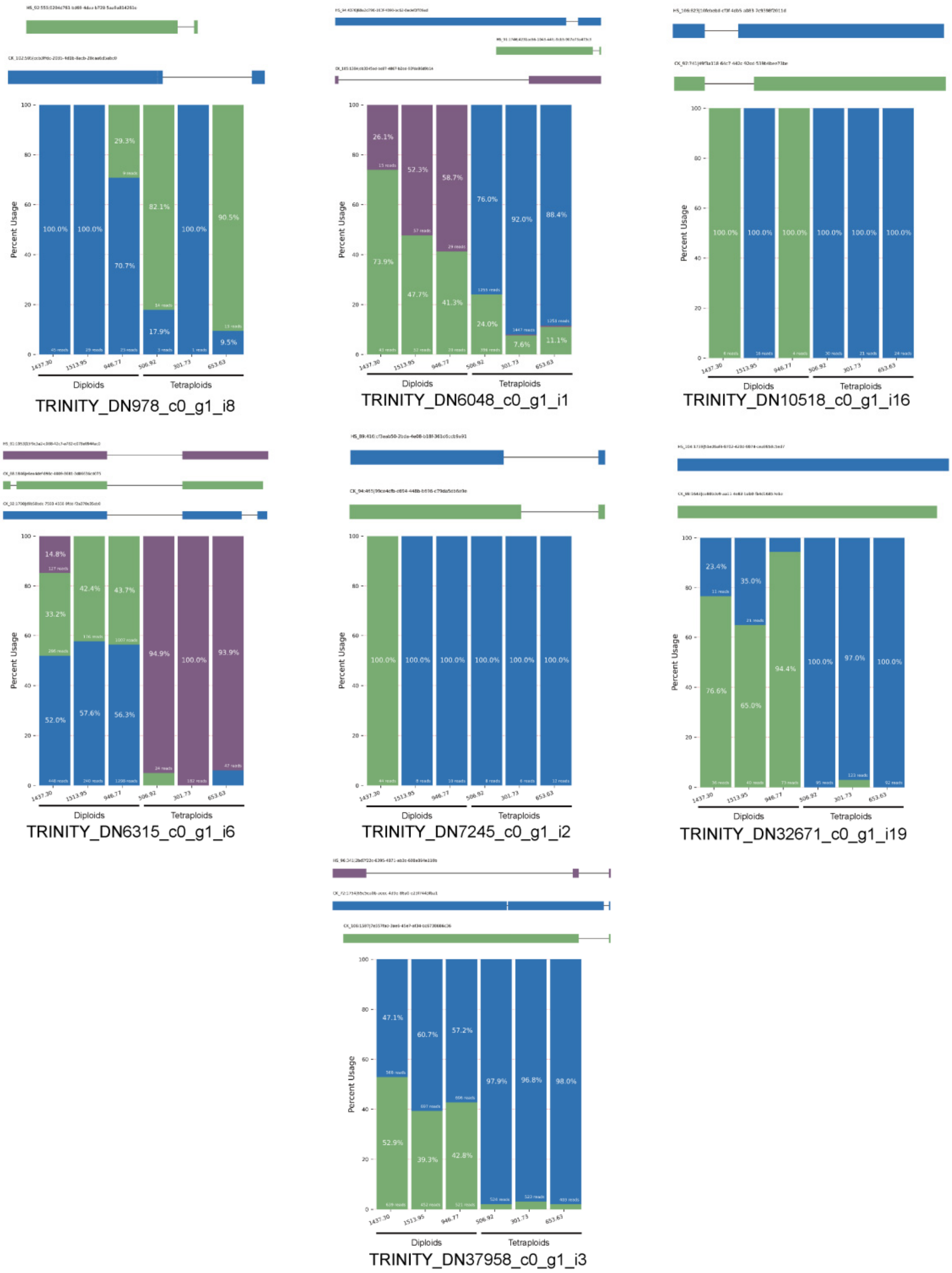

Figure S23. Schematic structure (top) and expression of isoforms based on RNA-seq (bottom) of the DSGs related to flowering and flower development.
